# Identification and Characterization of Post-activated B Cells in Systemic Autoimmune Diseases

**DOI:** 10.3389/fimmu.2019.02136

**Published:** 2019-09-24

**Authors:** Sarah Y. Weißenberg, Franziska Szelinski, Eva Schrezenmeier, Ana-Luisa Stefanski, Annika Wiedemann, Hector Rincon-Arevalo, Anna Welle, Annemarie Jungmann, Karl Nordström, Jörn Walter, Juliana Imgenberg-Kreuz, Gunnel Nordmark, Lars Rönnblom, Prathyusha Bachali, Michelle D. Catalina, Amrie C. Grammer, Peter E. Lipsky, Andreia C. Lino, Thomas Dörner

**Affiliations:** ^1^Department of Rheumatology and Clinical Immunology, Charité University Medicine Berlin, Berlin, Germany; ^2^German Rheumatism Research Center Berlin (DRFZ), Berlin, Germany; ^3^Grupo de Inmunología Celular e Inmunogenética, Facultad de Medicina, Instituto de Investigaciones Médicas, Universidad de Antioquia UdeA, Medellín, Colombia; ^4^Department of Genetics and Epigenetics, Saarland University, Saarbrücken, Germany; ^5^Department of Medical Sciences, Rheumatology and Science for Life Laboratory, Uppsala University, Uppsala, Sweden; ^6^RILITE Research Institute, Charlottesville, VA, United States

**Keywords:** systemic lupus erythematosus, rheumatoid arthritis, primary Sjögren's syndrome, B cell receptor signaling, toll-like receptor 9, CD40, post-activation, anergy

## Abstract

Autoimmune diseases (AID) such as systemic lupus erythematosus (SLE), primary Sjögren's syndrome (pSS), and rheumatoid arthritis (RA) are chronic inflammatory diseases in which abnormalities of B cell function play a central role. Although it is widely accepted that autoimmune B cells are hyperactive *in vivo*, a full understanding of their functional status in AID has not been delineated. Here, we present a detailed analysis of the functional capabilities of AID B cells and dissect the mechanisms underlying altered B cell function. Upon BCR activation, decreased spleen tyrosine kinase (Syk) and Bruton's tyrosine kinase (Btk) phosphorylation was noted in AID memory B cells combined with constitutive co-localization of CD22 and protein tyrosine phosphatase (PTP) non-receptor type 6 (SHP-1) along with hyporesponsiveness to TLR9 signaling, a Syk-dependent response. Similar BCR hyporesponsiveness was also noted specifically in SLE CD27^−^ B cells together with increased PTP activities and increased transcripts for *PTPN2, PTPN11, PTPN22, PTPRC*, and *PTPRO* in SLE B cells. Additional studies revealed that repetitive BCR stimulation of normal B cells can induce BCR hyporesponsiveness and that tissue-resident memory B cells from AID patients also exhibited decreased responsiveness immediately *ex vivo*, suggesting that the hyporesponsive status can be acquired by repeated exposure to autoantigen(s) *in vivo*. Functional studies to overcome B cell hyporesponsiveness revealed that CD40 co-stimulation increased BCR signaling, induced proliferation, and downregulated PTP expression (*PTPN2, PTPN22*, and receptor-type PTPs). The data support the conclusion that hyporesponsiveness of AID and especially SLE B cells results from chronic *in vivo* stimulation through the BCR without T cell help mediated by CD40–CD154 interaction and is manifested by decreased phosphorylation of BCR-related proximal signaling molecules and increased PTPs. The hyporesponsiveness of AID B cells is similar to a form of functional anergy.

## Introduction

Breach of self-tolerance, maintenance of autoimmune memory, and continuous autoantibody production are important pathologic features of B cells in autoimmune diseases (AID) (1) such as systemic lupus erythematosus (SLE), rheumatoid arthritis (RA), and primary Sjögren's syndrome (pSS). A number of phenotypic abnormalities of peripheral B cell subsets have been reported in AID (2–10), but their relationship to the functional abnormalities of the B cell axis has not been fully delineated. As antigen-induced B cell receptor (BCR) signaling is crucial for B cell fate (11–13), the BCR plays a pivotal role in the development and maintenance of autoimmunity. Therefore, BCR signaling has been extensively studied and there is general consensus that pathologically increased BCR signaling contributes to B cell overactivity and autoimmunity. This was the rationale for testing the impact of the anti-CD22 monoclonal antibody, epratuzumab, in SLE, since it was thought that CD22 engagement would impose negative regulation of BCR signaling (14). Since this trial failed to meet its primary endpoint (15), it is possible that hyperreactive BCR signaling is not central to SLE and perhaps other AID.

The BCR acts as a signal transducer, integrating receptor occupancy to downstream events that regulate cellular survival and activation. The strength of the signal determines cell fate and is tightly balanced by the activities of stimulatory and inhibitory molecules, including various phosphotyrosine kinases (PTKs) and phosphotyrosine phosphatases (PTPs) (11–13). Antigen binding induces phosphorylation of the BCR-associated Igα (CD79a) and Igβ (CD79b) chains leading to downstream Lyn and spleen tyrosine kinase (Syk) phosphorylation (16). This activates 1-phosphatidylinositol-4,5-bisphosphate phosphodiesterase gamma-2 (PLCγ2), Bruton's tyrosine kinase (Btk), and protein kinase B (Akt), which results in Ca^2+^- and Akt-dependent transcription (17–19). As a negative feedback loop, e.g., CD22 becomes phosphorylated and recruits PTP non-receptor type 6 (SHP-1) to the BCR, which dephosphorylates BCR downstream targets (14, 20, 21).

Genome-wide association studies (GWAS) identified polymorphisms of BCR downstream scaffold proteins, PTKs and PTPs, as associated with AID, supporting the idea of intrinsically defective BCR signaling in AID (22–29). Despite these suggestions, the major understanding of BCR signaling in autoimmunity is mainly based on studies in mice, in which BCR hyperreactivity can be a main driver of autoimmunity (30). However, in humans, the data are contradictory. There are studies reporting increased BCR signaling measured by Ca^2+^ release and downstream tyrosine phosphorylation related to a lack of negative regulation, such as by low-affinity immunoglobulin gamma Fc receptor II-b (FcγRIIb), phosphatase and tensin homolog (PTEN), or Lck/Yes novel tyrosine kinase (Lyn), in B cells from SLE patients (31–37). In contrast, a growing body of literature provides evidence that the BCR signal in autoimmunity is impaired, at least in some B cell subsets, with reduced tyrosine phosphorylation, Ca^2+^ release, and recruitment of signaling kinases to lipid rafts upon BCR stimulation (38–41).

In addition to abnormalities in the BCR pathway of stimulation, T cell-independent responses, such as TLR9 responses, have been reported to enhance B cell activation, in particular when autoantigen/ribonucleoprotein-immune complexes simultaneously engage BCR and TLR9 (42). However, recent reports indicate that SLE B cells also display low reactivity following TLR9 signaling (43–45). In addition to intrinsic B cell abnormalities, alteration of the functional status of other cells involved in regulating antibody production may also contribute to the development of AID. In this regard, abnormal germinal center (GC) reactions in autoimmune tissues (46), disturbances of regulatory T cells (1), increased T_FH_ (47), abnormalities of CD4^+^ (48), and CD8^+^ T cells (49, 50) with diminished T cell responses have also been reported. Therefore, the precise set of abnormalities underlying B cell dysfunction in AID remains to be fully delineated.

Here, we carried out a comparative analysis of peripheral as well as tissue-resident B cells from different AID patients and demonstrate that AID B cells share a phenotype of hyporesponsiveness toward BCR and TLR9 stimulation. This suggests a common signaling dysfunction between these diseases. However, SLE B cells appear to display a more prominent phenotype as not only antigen experienced conventional CD27^+^ memory but also CD27^−^ B cells exhibit BCR dysfunction. Of note, within this study, we compare characteristics of CD27^−^ and CD27^+^ B cells that can also include CD27^−^ isotype switched B cells or switched and non-switched CD27^+^ memory B cells (7, 8, 51, 52). The composition of CD27^−^ and CD27^+^ B cell subsets may differ among AID patients and healthy donors (HD) (53). Therefore, analyzing CD27^−^ and CD27^+^ subsets as a whole prevented us from determining whether there were subtle changes in different subsets. This decreased responsiveness, likely induced by chronic BCR engagement *in vivo*, can be partially overcome by CD40 engagement, which reduced the expression of PTPs, such as *PTPN22*. Therefore, the functional B cell anergy detected *in vitro* in AID B cells appears to reflect intensive BCR engagement *in vivo*.

## Materials and Methods

### Donors

EDTA anticoagulated peripheral blood samples were obtained from 85 SLE, 42 RA, and 51 pSS patients and 118 HD. Donor details are listed in Table S1. Patients with RA fulfilling the ACR/EULAR criteria (54), SLE meeting the SLICC criteria (55), and pSS fulfilling the AECG criteria (56) were included in this study. Tissue samples were obtained from surgeries: four spleen samples from patients with immune thrombocytopenia (ITP), seven spleen samples and four tonsil samples from patients without autoimmune background, and one parotid sample from a pSS patient. All patients and donors gave their consent according to the approval of the local ethics' committee at the Charité University Hospital Berlin. Written consensus was obtained from all patients and controls.

### Antibodies and Reagents

Staining antibodies, stimulation reagents, media, and other reagents were purchased from BD Bioscience (Franklin Lakes, NJ, USA), BioLegend (San Diego, CA, USA), eBioscience/Thermo Fisher (Carlsbad, CA, USA), Illumina (San Diego, CA, USA), Invitrogen/Thermo Fisher (Carlsbad, CA, USA), Life Technologies/Thermo Fisher (Carlsbad, CA, USA), Miltenyi Biotec (Bergisch Gladbach, Germany), Jackson ImmunoResearch (West Grove, FL, USA), PeproTech (Rocky Hill, NJ, USA), Promega Corporation (Madison, WI, USA), Selleck Chemicals (Houston, TX, USA), Sigma-Aldrich (St. Louis, MO, USA), and UCB Pharma (Slough, UK) and listed in Tables S2, S3. Quality control of flow cytometry stainings was performed using SPHERO Rainbow Calibration Particles (BD Bioscience, Franklin Lakes, NJ, USA) and Cytometer Setup and Tracking beads (BD Biosciences, Franklin Lakes, NJ, USA) for stable MFIs over time (57).

### Whole Blood Analysis for Intracellular Phenotyping

Fresh peripheral whole blood (100 μl) was lysed and fixed in 1 ml pre-warmed Lyse/Fix buffer (BD Bioscience) for 10 min at 37°C. Permeabilization and staining were performed as previously described (40). Cells were stained with anti-CD3, -CD14, -CD19, -CD20, and -CD27 or combinations of anti-protein and anti-phospho-protein antibodies: Syk/pSyk(Y^352^), Akt1/pAkt(S^473^), Btk/pBtk(Y^223^), and PLCγ2/pPLCγ2(Y^759^), respectively. Flow cytometry analysis was performed using a FACSCanto II flow cytometer (BD Bioscience Franklin Lakes, NJ, USA). The gating strategy used is shown in Figure S1. As an internal negative control, CD3^+^ T cells were included for all protein and phospho-protein antibodies assays except Akt1, where an isotype control was conducted, as Akt1 is widely expressed among lymphocytes.

### Isolation of Peripheral Blood Mononuclear Cells (PBMCs)

PBMCs were isolated using density gradient centrifugation method as described previously (40). Freshly isolated cells were directly suspended in ice-cold MACS rinsing buffer (with BSA; Miltenyi) for B and T cell purification, in pre-warmed RPMI 1640 (with GlutaMAX, Life Technologies) for short-term stimulation assays, or in pre-warmed phosphate buffered saline (PBS) for carboxyfluorescein succinimidyl ester (CFSE, Invitrogen/Thermo Fisher, Carlsbad, CA, USA) staining and long-term *in vitro* culture. Cells from at least one HD and one patient were analyzed simultaneously to enhance reliability.

### Isolation of Mononuclear Cells (MNCs) From Tissues

MNCs from tissues were isolated from spleens, tonsils, and parotid as described previously (58). Cells were released from minced tissue samples by shaking with ice-cold MACS buffer. Samples were filtered (70 μm cell strainer, Corning, NY, USA) and MNCs were isolated using density gradient centrifugation. Residual erythrocytes were removed using EL Buffer (Quiagen, Venlo, Netherlands). Cells were stored at −20°C within FBS/DMSO buffer.

### B and T Cell Enrichment

B and T cell enrichment from PBMCs was carried out using human B cell Kit II or human Pan T cell kit (Miltenyi Biotec, Bergisch-Gladbach, Germany) for magnetic cell sorting according to the manufacturer's protocols. B and T cell purities were checked by flow cytometry after staining with anti-biotin and anti-CD19 or anti-CD3 antibodies. Cell suspensions with 82% purity were used for further experiments.

### Determination of PTP and Protein Serine/Threonine Phosphatase (PSP) Activities

Purified B or T cells were lysed for 30 min on ice with Halt Protease Inhibitor Cocktail (1% in Pierce IP Lysis Buffer; Thermo Fisher). Then, the assay was processed according to the manufacturer's protocol and as described previously (59) using a commercial PTP and protein serine/threonine phosphatase (PSP) activity kit (Promega Corporation); 25,000 cells/well (PTP) and 80,000 cells/well (PSP) were used. In order to ensure the specificity of the PTPs and PSP activity, the same experiments were performed using the inhibitors monovanadate (10 mM) and sodium fluoride (10 mM) (Sigma-Aldrich), respectively. Cell lysates were analyzed at 600 nm using a Spectramax Plus 384 micro plate reader (Molecular Devices, San Jose, CA, USA). Phosphatase activity was quantified by the release of free phosphate. Concentrations were assessed from standard dilution series.

### BCR-Associated Protein Kinase Phosphorylation Kinetics Using Phosflow (BD Bioscience)

For functional phosphorylation kinetics, PBMCs or thawed MNCs (10^6^ cells) were rested for 1 h at 37°C in RPMI and stimulated with anti-IgG/IgM F(ab′)_2_ (13 μg/ml) for 2, 5, 8, 15, and 30 min, respectively. An additional RPMI control served as control at baseline. BCR stimulation was stopped by adding 1 ml of pre-warmed Lys/Fix buffer (BD Bioscience). Lysis, fixation, permeabilization, and staining were performed as described previously (40). Cells were stained with anti-CD3, -CD14, -CD19, -CD20, -CD27, and combinations of Syk/pSyk(Y^352^), Syk/pAkt(S^473^), or Btk/pBtk(Y^223^), respectively. Flow cytometry analysis was performed using a FACSCanto II flow cytometer. MFIs were used to assess phosphorylation intensity of phospho-proteins within different B cell subsets (gating strategy see Figure S1). Previously reported CD27^−^Syk^++^ cells (60) were excluded in pSyk(Y^352^) and pAkt(S^473^) kinetics, because they have been shown to represent a population of memory-like B cells.

### Chronic BCR Stimulation and CD40 Co-stimulation

For chronic BCR stimulation experiments, cells were pre-incubated with anti-IgG/IgM (2 μg/ml), CpG (0.5 μg/ml) or RPMI for 24, 48, or 72 h (37°C, 5% CO_2_) and subsequently stimulated with anti-IgG/IgM or RPMI as a control for 5 min. For co-stimulation experiments, cells were pre-incubated with recombinant human IL-4 (20 ng/ml) or IL-21 (20 ng/ml) or CD40L (500 ng/ml, human CD40L Multimer kit, Miltenyi Biotec) or combinations thereof for 48 h (37°C, 5% CO_2_) and subsequently stimulated with anti-IgG/IgM or RPMI as a control for 5 min. Cells were lysed, fixed, permeabilized, stained for Syk/pSyk(Y^352^), and analyzed as described above. Flow cytometry analysis was performed using a FACSCanto II or LSRFortessa flow cytometer.

### CD22/SHP-1 Co-localization Analysis

Purified B cells were suspended in 100 μl of RPMI (0.2 × 10^6^ cells) equilibrated for 1 h at 37°C, stimulated with anti-CD22 F(ab′)_2_ epratuzumab-A488 (10 μg/ml) (UCB) or left unstimulated, fixed, and permeabilized as described above. Cells were stained with mouse-anti-human-SHP-1 (BD Bioscience) and, for unstimulated control, with anti-CD22 F(ab′)_2_ epratuzumab-A488. Washed cells were stained with the secondary antibodies donkey-anti-mouse-RRX. Finally, cells were centrifuged onto slides and covered with Vectashield HardSet mounting medium containing 4′,6-diamidino-2-phenylindole (DAPI) (Vector Laboratories, USA) to stain the nucleus. Co-localization/fluorescence overlap of SHP-1/CD22 was evaluated (0 = no co-localization, 1 = all pixels co-localized) using a Nikon A1-Rsi confocal microscope and NIS Elements C imaging software (Nikon, Tokyo, Japan).

### Proliferation Studies Using CFSE

For cell proliferation analysis upon B cell stimulation, PBMCs (2 × 10^6^ cells/ml in PBS) were stained with CFSE (5 μM; Invitrogen) for 4 min at 37°C prior stimulation. After staining, cells were washed and incubated with 10 ml of RPMI for 30 min at 37°C. Before proceeding to the stimulation protocol, cells were washed with RPMI (10% FBS, 1% P/S).

### *In vitro* B Cell Differentiation

PBMCs (10^6^ cells per well) were rested for 1 h at 37°C and subsequently stimulated with CpG (0.5 μg/ml), anti-IgG/IgM (2 μg/ml), or CD40L (500 ng/ml) and the combinations CpG/CD40L, CpG/anti-IgG/IgM, or CpG/anti-IgG/IgM/CD40L for 5 days (37°C, 5% CO_2_) in RPMI (10% FBS, 1% P/S). For B cell proliferation, recombinant human IL-2 (20 ng/ml) and IL-10 (20 ng/ml) (Miltenyi Biotec) were added to the culture. After stimulation, cells were washed with MACS buffer and stained for 15 min at 4°C. Cells were stained with CD3, CD14, CD19, CD20, CD27, and CD38. Before flow cytometry analysis, DAPI was added to exclude dead cells. Cells were analyzed using a FACSCanto II flow cytometer (BD Bioscience; see gating strategy on Figure S5).

### Analysis of Differentially Methylated CpGs

Idat files of the Illumina Infinium HumanMethylation450 BeadChip data (61–63) were processed with RnBeads (v1.6.1). Data were aligned to hg 19 reference genome, and SNPs and sex chromosomes were excluded from further processing. A bead count cutoff of ≥10 and a greedy cut *p*-value cutoff of ≤ 0.01 was set to filter the data for high quality. The data were normalized by using the swan method. DMCs were defined as CpGs with ≥5% DNA methylation difference and *p* ≤ 0.01.

### RNA Sequencing Analysis of CD40/IL-4R-Stimulated B Cells

Isolated PBMCs were cultured overnight with CD40L (500 ng/ml) and IL-4 (20 ng/ml) or medium as a control. FcR blocking reagent was added to cell suspension before cells were stained with anti-CD14, -CD27, -CD20, -CD19, -CD3, -CD16, and DAPI, and CD19^+^ B cells were sorted directly into Arcturus PicoPure Extraction Buffer (Thermo Fisher) using a FACSAria™ II Sorter (BD Bioscience). Stranded sequencing library preparation of total mRNA was done using TruSeq RNA library prep kit (Illumina). Around 60 million passed filter reads per library were collected in a paired end read mode with 50-bp sequences each by the HiSeq2500 Illumina system.

### Differential Gene Expression Analysis of RNA-Seq Data

RNA-Seq from stimulated and control CD19^+^ B cells was performed on two SLE and one HD and deposited under PRJNA564980 at NCBI sequence read archive. Three technical replicates were included for each cohort and time point; files were obtained from FASTQC. FASTQC, Trimmomatic, STAR, Sambamba, and featureCounts were done separately. After careful examination of the PCA plots, three technical replicates of each cohort and condition were averaged into one and then used to perform relative gene expression. After FASTQC quality control analysis, Trimmomatic was used to cut adapter sequences, low-quality reads, and the first 14 reads of each sequence due to non-random primer bias. Reads were aligned to the human reference genome hg38 in STAR, and the.sam files were converted to sorted.bam files using Sambamba. Relative DE counts were generated in featureCounts. FastQC, Trimmomatic, STAR, Sambamba, and the featureCounts programs are all free, open source programs available at the following web addresses:

FastQC—https://www.bioinformatics.babraham.ac.uk/projects/fastqc/
Trimmomatic—http://www.usadellab.org/cms/?page$=$~trimmomatic
STAR—https://github.com/alexdobin/STAR
http://labshare.cshl.edu/shares/gingeraslab/www-data/dobin/STAR/STAR.posix/doc/STARmanual.pdf
Sambamba—http://lomereiter.github.io/sambamba/
FeatureCounts—http://subread.sourceforge.net/

### Differential Gene Expression of Publicly Available SLE and MS Data Sets

Data were derived from publicly available data sets: GSE4588 CD20^+^ B cells from SLE and HD (6 SLE, 7 HD), GSE117935 CD19^+^ B cells from multiple sclerosis (MS) patients and HD (10 MS, 10 HD), E-MTAB-2713 CD4^+^ T cells from SLE and HD (53 SLE, 41 HD), and E-MTAB-2713 CD8^+^ T cells from SLE and HD (22 SLE, 31 HD). DE was done for each data set of SLE patients and HD. GCRMA normalized expression values were variance corrected using local empirical Bayesian shrinkage before calculation of DE using the ebayes function in the open source package BioConductor LIMMA package (https://www.bioconductor.org/packages/release/bioc/html/limma.html). Resulting *p*-values were adjusted for multiple hypothesis testing and filtered to retain DE probes with an FDR < 0.2 (64).

### Data Analysis and Statistics

All flow cytometry data were analyzed with FlowJo (version 10.3, TreeStar, Ashland, OR. USA). Statistical analysis was performed with GraphPad Prism (version 5.04, GraphPad Software, La Jolla, CA. USA). For all data sets, Gaussian distribution was assumed. For the comparison of two groups, unpaired *t*-test was applied, and for paired analysis, paired *t*-test was applied. When multiple groups were compared, one-way ANOVA with Dunnett's test for multiple comparisons (DMCT) was applied. For the comparison of time-dependent kinetics and multiple groups, two-way ANOVA with Bonferroni test for multiple comparisons (BMCT) was used.

## Results

### Enhanced PTP Activity by SLE CD19^+^ B Cells

**Figure 1:**
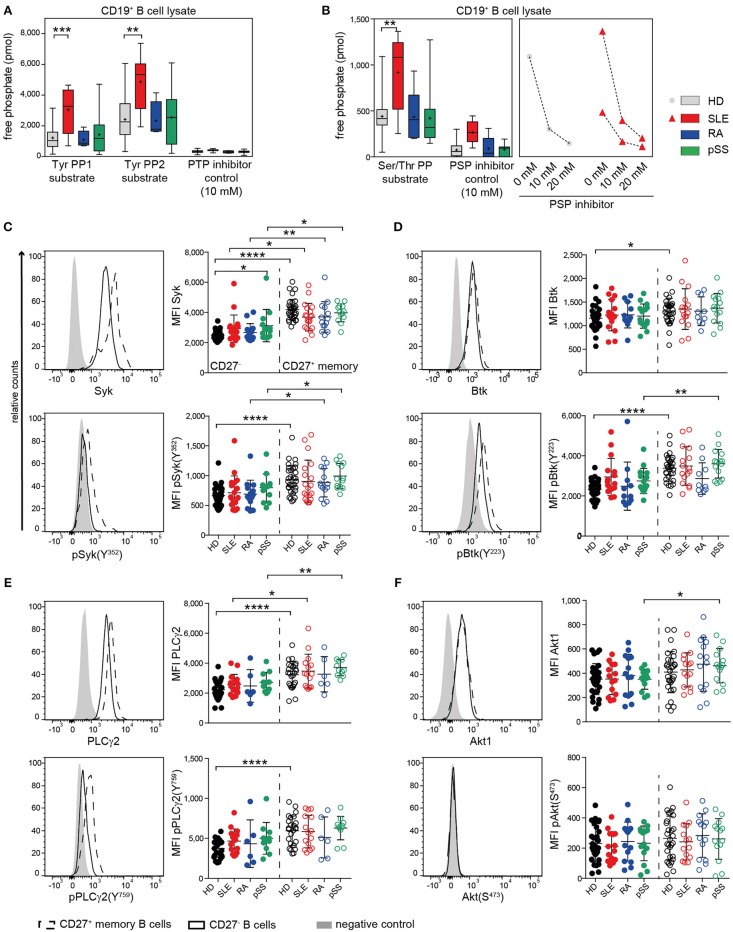
Enhanced PTP activity in SLE B cells and similar kinase expression and phosphorylation in AID among CD27^−^ and CD27^+^ memory B cells. **(A)** PTP [n(HD/SLE/RA/pSS) = 22/8/4/13] and **(B)** PSP [n(HD/SLE/RA/pSS) = 19/8/5/10] activities of CD19^+^ B cells in HD (gray), SLE (red), RA (blue), and pSS (green). CD27^−^ (dots) and CD27^+^ memory (open circles) B cells from HD (black), SLE (red), RA (blue), and pSS (green) patients were analyzed for the expression of **(C)** Syk/pSyk(Y^352^) [n(HD/SLE/RA/pSS) = 32/19/14/13]; **(D)** Btk/pBtk(Y^223^) [n(HD/SLE/RA/pSS) = 30/16/11/15]; **(E)** PLCγ2/pPLCγ2(Y^759^) [n(HD/SLE/RA/pSS) = 25/15/6/11] and **(F)** Akt1/pAkt(S^473^) [n(HD/SLE/RA/pSS) 30/14/14/13]. Representative histograms of CD27^−^ (solid line); CD27^+^ (dashed line), and negative control (gray) are shown. Box whisker plots represent median (line), mean (plus), and the range from minimum to maximum; lines in scatter dot plots represent means ± SD (ANOVA with DMCT; *t*-test; ^*^p ≤ 0.05, ^**^p ≤ 0.01, ^***^p ≤ 0.001, ^****^p ≤ 0.0001).

BCR signaling is regulated by a finely tuned balance of PTKs and PTPs (16, 65). In a previous study, we found that B cells from SLE patients have enhanced PTP activities and reduced Syk phosphorylation compared to HDs (40). Most SLE patients exhibited this abnormality, independent of disease activity. To determine whether these abnormalities are unique to SLE, we assessed PTP and PSP activities in SLE, RA, pSS, and HD CD19^+^ B cells (Figure 1A). SLE B cells exhibited significantly increased PTP activities for both PTP-specific substrates (TyrPP1 and TyrPP2), whereas no significantly increased PTP activities were found in RA and pSS B cells (Figure 1A). Similarly, we found that PSP activity was uniquely increased in SLE, but not in RA or pSS patients compared to HD. We employed specific PTP and PSP inhibitors to ensure specificity of the PTPs/PSPs analyzed. PTP activity could be blocked and PSP inhibition was dose dependent (Figures 1A,B). PTP and PSP activities in CD3^+^ T cells from AID patients were similar to HD (Figure S1), suggesting that enhanced PTP/PSP activity is a unique characteristic of SLE B cells.

The location of a PTP, such as SHP-1, is important for BCR signaling regulation. Here, we analyzed the co-localization of SHP-1 (*PTPN6*) with CD22 with and without anti-CD22 engagement, which recruits and activates SHP-1. Cap formation was not confined to one or the other AID, with increased baseline co-localization of SHP-1 and CD22 in SLE, pSS, and RA patients. Notably, the degree of co-localization constitutively present in SLE B cells appeared at a maximum and could not be further increased upon CD22 engagement. In contrast, CD22 engagement increased co-localization of SHP-1 in RA, pSS, and HD B cells (Figure S1). The data suggest that a functionally active PTP complex of SHP-1 was substantially increased in SLE B cells at baseline.

### Similar Baseline Expression and Phosphorylation Levels of BCR-Associated Protein Kinases and PLCγ2 in HD and AID Patients

Next, we studied the status of BCR downstream kinases and phospholipase PLCγ2 in CD27^−^ and CD27^+^ memory B cells of AID patients. We analyzed the baseline phosphorylation of Syk, Btk, and PLCγ2, which are regulated by PTPs. Furthermore, we analyzed Akt1 regulated by PSPs. We found similar basal Syk expression and phosphorylation at the activation site Syk(Y^352^) within CD27^−^ and CD27^+^ memory B cells in patients and HD (Figure 1C). Syk expression and baseline phosphorylation were generally higher in CD27^+^ memory than in CD27^−^ B cells across all groups. For the downstream signaling molecules Btk and PLCγ2 and their phosphorylation sites Btk(Y^223^) and PLCγ2(Y^759^), we found similar expression and phosphorylation levels among all patients and HD (Figures 1D,E). Akt1 expression and phosphorylation at Akt(S^473^) were comparable among CD27^−^ and CD27^+^ B cells from all donor groups (Figure 1F). Constitutive expression and phosphorylation levels of BCR-associated kinases (Figure 1) were overall similar to HD in all AID tested in both B cell subsets, despite increased constitutive phosphatase activity in SLE B cells.

### Reduced Syk and Btk Tyrosine Phosphorylation Upon BCR Stimulation Is Characteristic of CD27^+^ Memory B Cells From Patients With AID and CD27^−^ SLE B Cells

**Figure 2:**
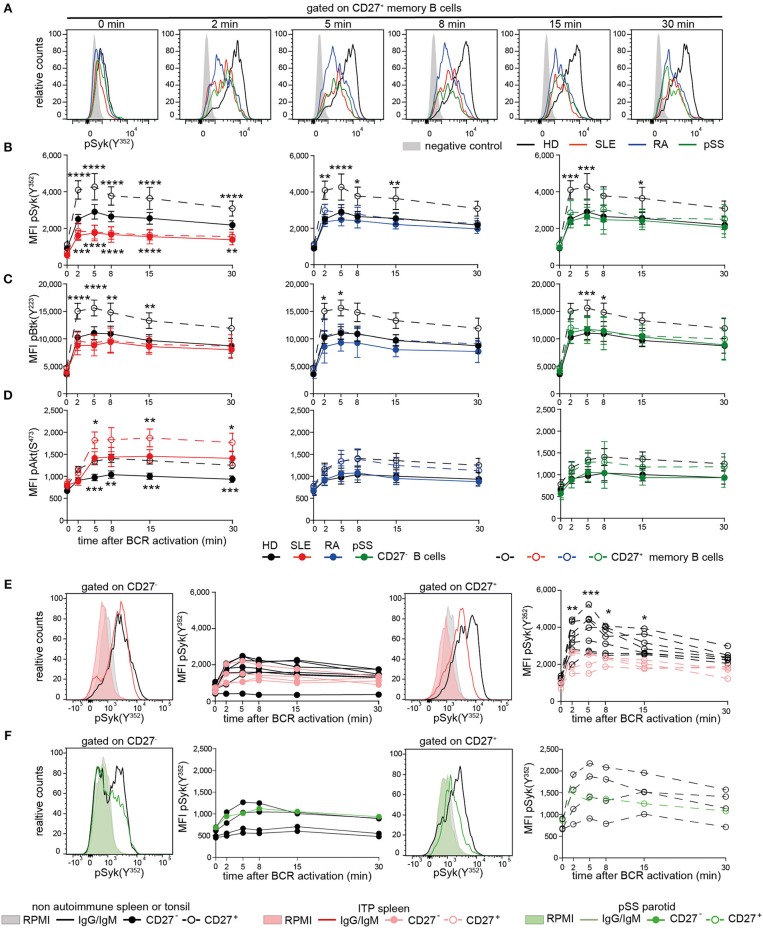
Reduced PTK phosphorylation upon BCR signaling in peripheral and tissue-resident CD27^+^ B cells from patients with AID. PBMCs or MNCs from HD (black), SLE (red), RA (blue), pSS (green), and ITP (pink) vs. non-ITP (black) spleens and pSS parotid gland (green) vs. tonsils (black) were stimulated with anti-IgG/IgM F(ab)_2_. **(A)** Representative pSyk(Y^352^) histograms from CD27^+^ memory B cells are shown. CD3^+^ T cells served as control (gray). Phospho-kinetics of **(B)** Syk(Y^352^) [n(HD/SLE/RA/pSS) = 19/11/11/10], **(C)** Btk(Y^223^) [n(HD/SLE/RA/pSS) = 21/10/6/14], and **(D)** Akt(S^473^) [n(HD/SLE/RA/pSS) = 29/15/11/10] in CD27^+^ memory (dashed lines) and CD27^−^ (solid lines) B cells are shown. Phospho-Syk(Y^352^) kinetics in B cells from **(E)** spleen and **(F)** tonsil/parotid with representative histograms [n_spleens_(non-ITP/ITP) = 7/4; n(tonsils/parotid) = 4/1]. Histograms show unstimulated (filled areas) vs. stimulated (solid lines) cells. Data show mean ±95% CI (two-way ANOVA with BMCT for the comparison of AID vs. HD CD27^−^ or CD27^+^ memory B cells, respectively; ^*^p ≤ 0.05, ^**^p ≤ 0.01, ^***^p ≤ 0.001, ^****^p ≤ 0.0001).

Subsequently, we assessed phosphorylation of Syk(Y^352^), Btk(Y^223^), and Akt(S^473^) following BCR engagement in AID and HD CD27^−^ and CD27^+^ memory B cells (Figure 2, Figure S2). Even though the composition of switched and non-switched cells among CD27^−^ and CD27^+^ B cells may differ among patients and HD, we did not observe substantial heterogeneity on the functional level, except within the recently reported CD27^−^Syk^++^ cells, which were excluded from this analysis.

BCR-induced phospho-(p)Syk(Y^352^) in AID CD27^+^ memory B cells and SLE CD27^−^ B cells was significantly lower compared to HDs (Figures 2A,B). Btk(Y^223^) phosphokinetics were qualitatively similar to that of pSyk(Y^352^), with a maximum phosphorylation after 5 min (Figure 2C). AID CD27^+^ memory B cells revealed significantly reduced pBtk(Y^223^) in comparison to HDs, whereas Btk(Y^223^) phosphorylation was reduced in SLE and RA CD27^−^ B cells. SLE B cells do not differ in surface IgG and IgM expression compared to HD (40), indicating that this phenotype is not related to reduced Ig expression. Furthermore, we observed reduced phosphorylation in pSS patients who had not received any treatment, suggesting that these effects were not treatment dependent.

Akt(S^473^) phosphokinetics displayed a maximum after 8 min (Figure 2D). Phospho-Akt(S^473^) in both B cell compartments from RA and pSS patients was comparable to those from HDs, whereas SLE patients showed increased pAkt(S^473^) compared to HD, at all-time points. Of particular note, memory B cells from HD expressed higher levels of tested phospho-PTKs and pAkt(S^473^) than CD27^−^ B cells upon BCR engagement as a general characteristic.

Taken together, diminished pSyk(Y^352^) and pBtk(Y^223^), but not pAkt(S^473^), upon BCR stimulation is a common characteristic of AID memory B cells, whereas it is also present in SLE CD27^−^ B cells, suggesting that the SLE B cell compartment is more globally abnormal.

### Reduced Syk(Y^352^) Phosphorylation in CD27^+^ B Cells From AID Lymphoid Tissues

To determine whether these findings are restricted to circulating B cells, we analyzed tissue-resident CD27^−^ and CD27^+^ B cells from autoimmune and non-autoimmune patients (Figures 2E,F). ITP patients' spleens were compared to control spleens from non-autoimmune patients. Control tonsils were obtained from patients undergoing tonsillectomy. Parotid tissue was received from one pSS patient undergoing parotidectomy to exclude lymphoma.

We found reduced pSyk(Y^352^) kinetics in ITP CD27^+^ memory B cells compared to control spleens, whereas pSyk(Y^352^) kinetics in CD27^−^ B cells were comparable to controls (Figure 2E). Phospho-Syk(Y^352^) was also diminished in tonsillar and parotid B cells (Figure 2F). These data indicate that B cells with diminished BCR-induced PTK phosphorylation particularly in the CD27^+^ memory B cell compartment are present in tissues of AID patients and in the tonsil.

### Reduced Syk(Y^352^) Phosphorylation Is Acquired as a Result of Previous Stimulation of the BCR

**Figure 3:**
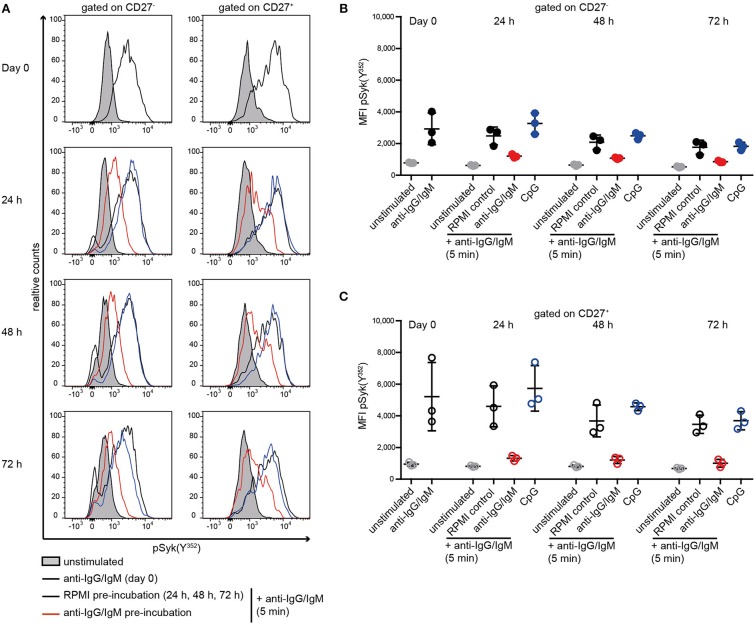
Reduced Syk(Y^352^) phosphorylation upon re-stimulation of the BCR. PBMCs from HD were incubated with anti-IgG/IgM, CpG, or RPMI as a control for 24, 48, or 72 h and re-stimulated with anti-IgG/IgM for 5 min. **(A)** Representative histograms of Syk(Y^352^) phosphorylation at day 0 and without (black line) or with anti-IgG/IgM (red) or CpG (blue) pre-incubation for 24, 48, or 72 h. The unstimulated control is shown as a gray peak. Anti-IgG/IgM induced pSyk(Y^352^) MFIs in **(B)** CD27^−^ B cells (solid dots) and **(C)** CD27^+^ B cells (open circles) at day 0 or after pre-incubation with RPMI as a control (black), anti-IgG/IgM (red), or CpG (blue) for 24, 48, and 72 h. Unstimulated controls are displayed as gray dots. Horizontal lines represent means ± SD [n(HD) = 3].

Subsequently, we tested whether reduced BCR signaling observed in SLE B cells and RA and pSS memory B cells can be induced in HD B cells by continuous stimulation with anti-BCR or TLR9 antagonists (Figure 3). Previously, we showed that *ex vivo* IgG and IgM expression does not differ between SLE and HD B cells (40). Here, continuous stimulation of HD B cells with anti-BCR, but not with CpG, resulted in reduced Syk(Y^352^) phosphorylation upon subsequent anti-BCR stimulation. This was observed for CD27^−^ and CD27^+^ memory HD B cells (Figures 3A–C).

### Co-stimulation With CD40L Normalizes BCR Responsiveness of B Cells From HD and AID Patients

**Figure 4:**
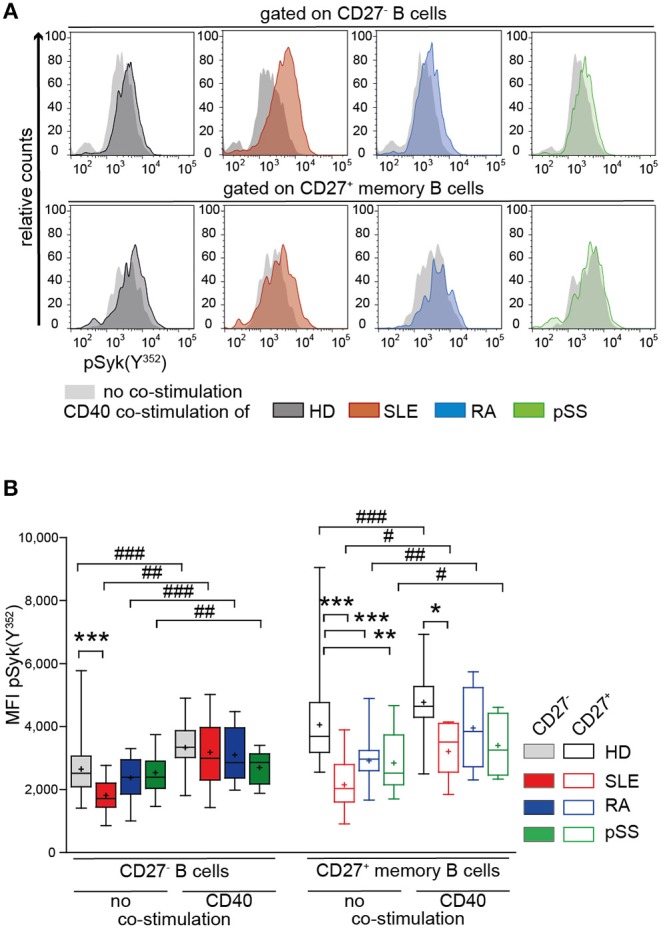
Increased Syk(Y^352^) phosphorylation reflecting BCR responsiveness of CD27^+^ memory B cells from HD, SLE, RA, and pSS upon co-stimulation with CD40L. PBMCs from HD (black), SLE (red), RA (blue), and pSS (green) patients were treated with CD40L and subsequently stimulated with anti-IgG/IgM F(ab)_2_. **(A)** Representative histograms of pSyk(Y^352^) in CD27^−^ (top row) and CD27^+^ memory (bottom row) B cells in the presence (colored areas) or absence (gray areas) of prior CD40L co-stimulation. **(B)** pSyk(Y^352^) in CD27^−^ (filled boxes) and CD27^+^ memory (clear boxes) B cells with [n(HD/SLE/RA/pSS) = 11/7/5/5] and without [n(HD/SLE/RA/pSS) = 30/18/16/15] CD40 co-stimulation. Box whisker plots represent median (line), mean (plus), and the range from minimum to maximum (ANOVA with DMCT, ^*^p ≤ 0.05, ^**^p ≤ 0.01, ^***^p ≤ 0.001; paired *t*-test, ^#^*p* ≤ 0.05, ^##^*p* ≤ 0.01, ^###^*p* ≤ 0.001).

Next, we tested the impact of CD40 co-stimulation on BCR-mediated signaling of AID B cells (66). We measured pSyk(Y^352^) upon CD40 co-stimulation followed by BCR engagement in CD27^−^ and CD27^+^ memory B cells from patients and HD (Figure 4).

Increased pSyk(Y^352^) was observed in CD27^−^ and CD27^+^ memory B cells from AID patients and HDs after treatment with CD40L compared to BCR stimulation alone (Figure 4A). After CD40L co-stimulation, Syk(Y^352^) phosphorylation in RA and pSS CD27^+^ memory B cells and in SLE CD27^−^ B cells was comparable to HD (Figure 4B), whereas BCR engagement alone gave rise to diminished pSyk(Y^352^) (Figures 2B, 4B). pSyk(Y^352^) expression by SLE CD27^+^ memory B cells increased upon CD40L co-stimulation compared to BCR stimulation alone, even though the phosphorylation amplitude remained lower than those in HD (Figure 4B). These data suggest improved BCR responsiveness by CD40L co-stimulation in memory B cells and in CD27^−^ SLE cells.

It has been reported that Th2 signals restore BCR signaling in a small population of anergic IgM^−^IgD^+^CD27^−^ B cells present in blood of HD and SLE patients (67). Therefore, we tested whether additional stimulation with IL-4 could further modulate pSyk(Y^352^) responses. IL-21, a key cytokine driving GC reactions (68), served as control. The presence of IL-4 or IL-21 alone led to modest effects on the pSyk(Y^352^) response to BCR engagement. IL-4 in combination with CD40L, however, led to higher responses than with CD40L alone in all groups, whereas the addition of IL-21 in combination with CD40L increased pSyk(Y^352^) only in CD27^−^ B cells in all donors (Figure S3).

We also tested whether co-stimulation affects basal expression and phosphorylation of Syk. We found that CD40L co-stimulation (with and without IL-4 or IL-21) caused a minor upregulation of Syk, whereas pSyk(Y^352^) was increased. SLE CD27^−^ B cells showed somewhat increased basal Syk and pSyk(Y^352^) upon CD40L co-stimulation with and without IL-4 or IL-21 (Figure S3). However, the amplitude of pSyk(Y^352^) changes upon CD40L stimulation was very modest.

### Diminished B Cell Proliferation and Antibody Secreting Cell (ASC) Differentiation Upon TLR9 Stimulation in Autoimmune Patients

**Figure 5:**
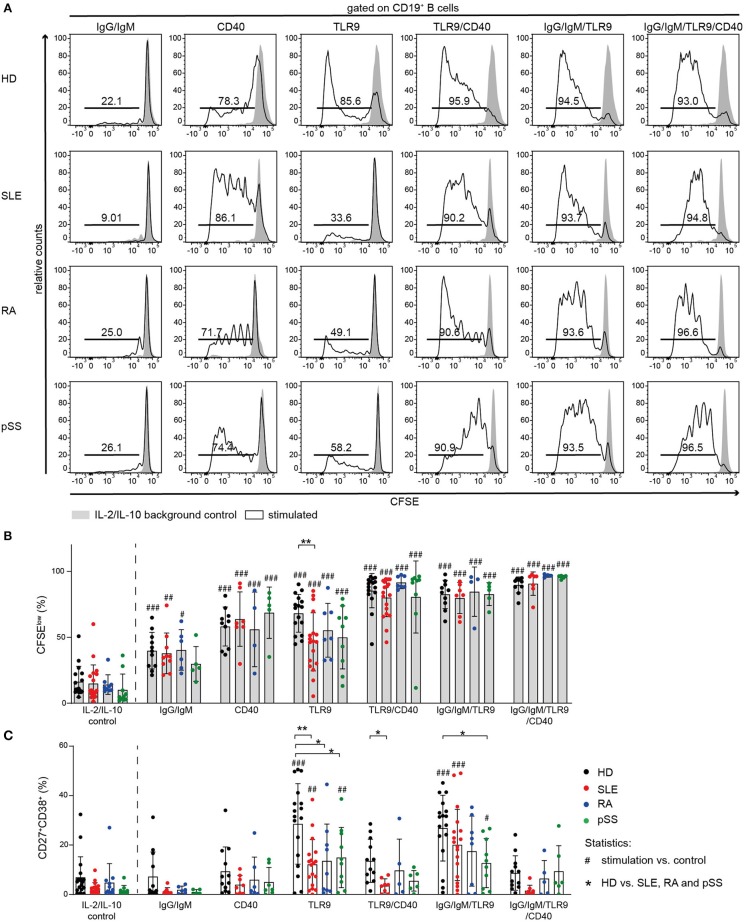
Proliferation of AID B cells upon CD40 stimulation or combined stimulation of BCR and TLR9 is comparable to HD. B cells from HD (black), SLE (red), RA (blue), and pSS (green) patients were stimulated with anti-IgG/IgM, CD40L, or CpG and the combinations CD40L/CpG, CpG/anti-IgG/IgM, or CD40L/CpG/anti-IgG/IgM. **(A)** Representative CFSE histograms of HD, SLE, RA, and pSS B cells after 5 days of culture for the indicated stimulus (lines) compared to background control (gray). **(B)** Percentages of CFSE^low^ B cells [n(HD/SLE/RA/pSS) = 17/19/9/10 (IL-2/IL-10 control); 11/10/6/5 (IgG/IgM); 10/7/4/5 (CD40); 16/18/7/9 (TLR9); 10/7/4/5 (TLR9/CD40); 16/19/7/9 (IgG/IgM/TLR9); 10/7/4/5 (IgG/IgM/TLR9/CD40)]. **(C)** Resulting frequency of CD27^+^CD38^+^ B cells upon activation in culture [n(HD/SLE/RA/pSS) = 18/18/11/10 (IL-2/IL-10 control); 12/8/6/5 (IgG/IgM); 12/8/7/7 (CD40); 17/18/9/10 (TLR9); 12/8/5/6 (TLR9/CD40); 17/18/8/10 (IgG/IgM/TLR9); 12/8/5/6 (IgG/IgM/TLR9/CD40)]. Bars shown represent mean ± SD (ANOVA with DMCT, ^*^p ≤ 0.05, ^**^p ≤ 0.01; ^#^*p* ≤ 0.05, ^##^*p* ≤ 0.01, ^###^*p* ≤ 0.001).

TLR9-mediated activation has been described to be impaired in SLE, including reduced production of cytokines upon TLR9 stimulation (43, 44). Thus, we tested whether reduced TLR9 responsiveness is a shared abnormality among AID B cells (Figure 5).

TLR9-induced B cell proliferation was decreased in AID B cells and diminished responsiveness was most pronounced in SLE B cells (Figures 5A,B). Moreover, TLR9-induced differentiation into ASCs was impaired in all AID B cells compared to HD (Figure 5C, Figure S4). Further, AID B cells gave rise to a twofold lower frequency of CD27^+^CD38^+^ cells compared to HDs when stimulated with CpG (Figure 5C, Figure S4).

It has been reported that Syk is necessary for TLR9 signaling (69, 70). To test whether Syk has a critical role in B cell activation following TLR9 stimulation, we stimulated HD B cells with CpG in the presence or absence of the Syk inhibitor entospletenib. Syk inhibition resulted in reduced frequencies of HD CD27^+^CD38^+^ B cells upon TLR9 stimulation, mimicking the B cell hyporesponsiveness of AID memory B cells, and SLE B cells (Figure S4).

Next, we investigated the effect of combined TLR9 and BCR stimulation because of the crucial role of TLR9 engagement in breaking tolerance against nuclear antigens and driving B cell activation (71–73). As both BCR and TLR9 appear to depend on Syk activity, combined stimulation increased differentiation of CD27^+^CD38^+^ ASC only modestly, but the response remained generally lower than HD B cell responses (Figure 5C). However, co-stimulation increased proliferation of all AID B cells to the level observed with HD B cells (Figure 5B).

### Co-stimulation With CD40L Results in Increased Proliferation of AID B Cells

Notably, CD40L activation induced B cell proliferation in all groups (Figure 5B). In line with previous reports (74), differentiation into CD27^+^CD38^+^ B cells was not observed upon stimulation with CD40L alone (Figure 5C, Figure S4).

In order to further investigate the effect of CD40 engagement on AID B cells and whether their hyporesponsiveness to TLR9 agonists can be overcome through CD40/CD40L interaction, we cultured AID and HD B cells in the presence or absence of CD40L together with CpG or the combination of CpG with anti-IgG/IgM (Figure 5).

Co-stimulation of CD40 increased TLR9-induced proliferation of AID B cells. Stimulation of AID B cells with CD40L and CpG compared to CpG alone resulted in increased frequencies of CFSE^low^ cells, similar to that induced in HD B cells (Figures 5A,B). However, the frequencies of CD27^+^CD38^+^ ASCs in CD40L co-stimulation cultures were lower compared to TLR9 or TLR9/BCR stimulation (Figure 5C) consistent with previous reports that CD40L stimulation blocks CpG induced *in vitro* B cell differentiation (74). Therefore, CD40 engagement provided the co-stimulation signal that allowed AID B cells, especially SLE B cells, to proliferate when co-stimulated through TLR9, but did not promote differentiation into CD27^+^CD38^+^ ASC.

### Common Methylation Pattern of B Cells From HD and AID Patients

Subsequently, we addressed whether reduced responsiveness of AID B cells is epigenetically controlled. Individual epigenome-wide association studies (EWAS) identified differentially methylated regions (DMR) in B cells from SLE (61), RA (63), and pSS patients (62) based on a meta-analysis of Infinium HumanMethylation450K BeadChip data of CD19^+^ cells.

The global methylation values of AID patients correlated strikingly with methylation values of HD samples (*r* = 0.99; Figure S5). We were interested in the methylation state of certain genes that are related to CD40 and BCR signaling, B cell activation, and, in addition, the methylation status of kinases and phosphatases (Figure S5). Except for the hypomethylation of multiple CpGs at the *IFITM1* locus, we could not detect substantial differences among selected CpGs. However, we found kinase *EIF2AK2* (cg14126601) and kinase modulator *TRIP6* (cg19279257) to be hypomethylated in AID.

On the global level, genes encoding interferon-induced proteins, such as *IFITM1, IFI44L*, and *MX1*, were found to be the most differentially methylated CpGs, with hypomethylation in all AID groups (Table S4). These genes are hypomethylated mainly in SLE and pSS and to a lesser extent in RA compared to HD (Figure S5).

### Decreased PTP Expression Upon CD40L/IL-4 Co-stimulation

Increased PTP/PSP activity was uniquely found in SLE CD19^+^ B cells (Figures 1A,B). Furthermore, we found that CD40/IL-4R co-signaling substantially improved BCR signaling (Figure S3). Based on this, we suggested that CD40/IL-4R signaling may be involved in the modulation of PTP and PSP expression. To test this hypothesis, we analyzed the expression of non-receptor (NR)- and receptor (R)-type PTPs and PSPs in SLE and the impact of CD40/IL-4R co-stimulation on their expression.

**Figure 6:**
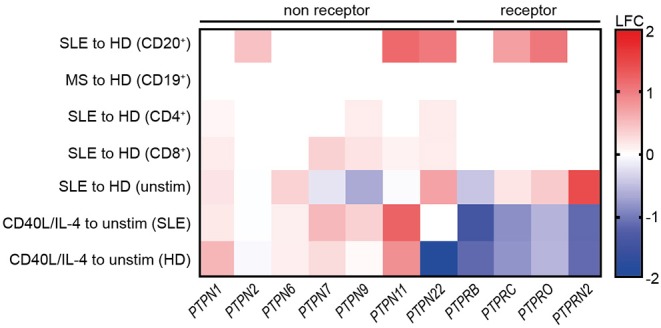
Common reduction of receptor-type PTP expression upon co-stimulation with CD40L/IL-4. Differential expression of selected genes related to receptor-type and non receptor-type PTPs in SLE vs. HD CD20^+^ B cells (6 SLE, 7 HD), MS vs. HD CD19^+^ B cells (10 MS, 10 HD), SLE vs. HD CD4^+^ (53 SLE, 41 HD), and CD8^+^ T cells (22 SLE, 31 HD) (lines 1–4 are from publicly available data). Differential gene expression from un-stimulated SLE vs. HD and CD40L/IL-4 stimulated for SLE vs. un-stimulated SLE or HD CD19^+^ B cells, respectively [n(HD/SLE) = 1/2], (lines 4–7).

First, we analyzed the expression of selected PTPs within publicly available gene expression data sets from SLE and HD CD20^+^ B cells, CD4^+^, and CD8^+^ T cells, and MS and HD CD19^+^ B cells as controls (Figure 6). Expression of *PTPN2, PTPN11, PTPN22, PTPRC*, and *PTPRO* was found to be specifically increased in SLE CD20^+^ B cells, as we found no differentially expressed PTP in MS B cells compared to HD. Further, the data point to a B cell-specific abnormality, as we found no differentially expressed RPTP and only marginal differential expressed NRPTP among CD4^+^ and CD8^+^ T cells from SLE compared to HD (Figure 6). These data correspond to the comparable PTP activity measured in SLE and HD CD3^+^ T cells (Figure S1).

Next, increased expression of *PTPN6* and *PTPRN2* in unstimulated SLE CD19^+^ B cells was detected. However, expression of *PTPRB* was decreased. Of note, *PTPN2* and *PTPN22* and all RPTP (except *PTPRB*) were downregulated upon CD40L/IL-4 stimulation compared to their overexpression before stimulation.

We tested the possibility that PSPs may be involved in abnormal SLE B cell signaling as we found increased PSP activity in SLE (Figure 1B). We analyzed genes related to the *PP2* and *PP2C* family. Unstimulated B cells displayed small differences in PSP expression between SLE and HD. CD40L/IL-4 co-stimulation led to heterogeneous changes (increased expression of PP2 family genes except *PPP2R3B* (unaffected) and decreased *PPP2R5D*, whereas PP2C family members were unaffected (Figure S6).

These data show that T cell help by CD40/IL-4R engagement alters the expression of NRPTPs such as *PTPN22* and different RPTPs and further underlines the crucial role of T cell co-stimulation in defining B cell dysfunction in SLE.

## Discussion

Despite information in the literature suggesting increased BCR signaling in AID (31, 37, 75), here we provide extensive evidence that BCR signaling and responses to TLR9 stimulation are reduced in AID memory B cells but more broadly in SLE B cells. Furthermore, it was demonstrated that this BCR signaling abnormality is not only characteristic of peripheral B cells, but is also present in tissue-resident B cells from ITP spleens and pSS parotid gland. In contrast to RA and pSS, in which BCR signaling defects are present only in antigen-experienced memory B cells, in SLE, this abnormality is also manifested in CD27^−^ B cells. Moreover, the SLE B cell abnormality extended to increased expression of PTPs, globally enhanced PTP and PSP activities, and maximal constitutive recruitment of the PTP SHP-1 to CD22. As a proof of concept, we compared PTP expression under stimulated and unstimulated conditions in two SLE and one healthy control subject. Although considered preliminary, results of this analysis were consistent with that obtained from larger publicly available data sets. Notably, CD40 co-stimulation resulted in normalization of BCR signaling, increased B cell proliferation, together with reduced expression of PTPs such as *PTPN22* in SLE. The B cell abnormalities are likely to reflect continuous stimulation through the BCR *in vivo* without appropriate co-stimulation. These results indicate that there is a spectrum of abnormalities in BCR signaling in AID, with SLE manifesting the most extensive B cell dysfunction and that the B cell dysfunction detected *ex vivo* and *in vitro* reflects varying degrees of BCR stimulation *in vivo*, without appropriate T cell-derived co-stimulation.

Reduced BCR signaling together with increased co-localization of the inhibitory co-receptor CD22 and SHP-1 in RA, pSS, and most pronounced in SLE B cells and increased PTP activities in SLE B cells may reflect a phenotype related to anergy (76–80). This conclusion is favored by the observation that repeated BCR engagement *in vitro* induces BCR hyporesponsiveness together with studies demonstrating that continuous signaling *via* SHP-1 is required to maintain anergy (80, 81). Moreover, the anergy phenotype extends to TLR9 signaling but does not involve the CD40 pathway. Finally, the anergy phenotype appears to be overcome by CD40 engagement, suggesting that it derives from incomplete signaling through the BCR *in vivo* without appropriate T cell-derived co-stimulation. Notably, the data suggest that when the proper series of signals are provided, AID B cells function in a largely normal manner, indicating that intrinsic B cell defects in these conditions may be minimal.

It has been reported that reduced signaling of phosphoinositide 3-kinase (PI3K) suppresses phosphatidylinositol (3,4,5)-trisphosphate (PIP3) generation by PI3K and is key for B cell anergy (82). In our studies, Akt signaling, which is downstream of PI3K, was not abnormal in RA and pSS, but was found to be increased in SLE. Another study demonstrated that proliferating GC B cells lack active BCR signaling which is induced and maintained by increased phosphatase activity and persistent co-localization of SHP-1 with the BCR after ligation (59). Therefore, reduced BCR signaling in peripheral blood and tissue-resident AID CD27^+^ B cells is a characteristic of what might be considered a post-activated functionally anergic phenotype. This status could be induced in HD B cells by chronic BCR stimulation, indicating that repetitive stimulation by self-antigens or immune complexes in the absence of appropriate co-stimulation can induce this functional status. Importantly, induction of this state of anergy appears to be specific for signaling through the BCR, as stimulation with CpG did not cause B cells to become anergic, even though anergic B cells exhibited decreased activation through TLR9.

Interestingly, reduced BCR-induced Syk phosphorylation in both CD27^−^ B cells and CD27^+^ memory B cells was uniquely found in SLE. Iwata et al. have previously reported increased phosphorylation of Syk in total SLE CD19^+^ compared to healthy controls (83). One difference to our data could be that Iwata et al. included in their analysis CD27^−^Syk^++^ memory-like B cells that are increased in SLE patients compared to HD (60). This population has been excluded in the current analysis. However, the more extensive abnormality appeared to be related to increased PTP activity and expression. This may reflect the more persistent stimulation of B cells in SLE, perhaps reflecting the enhanced expression of T follicular helper cells in SLE (47, 84) and recent findings that IL-21 promotes CD11c^hi^T-bet^+^ B cell development enriched of autoreactive cells in SLE (85). Even though increased T_FH_ and increased expression of CD40L have been reported in SLE, there may be compartmentalization of these cells away from sites of B cell activation, permitting persistent engagement of the BCR with appropriation T cell-derived T cell help.

Lymphocyte hyporesponsiveness in AID is not restricted to B cells. Impaired cytotoxic function and exhaustion of CD8^+^ T cells, a characteristic of viral infections, has been reported in SLE (49, 50, 86). SLE T cells display abnormal T cell signaling (48, 87) and reduced Th1/Th2 Ca^2+^ responses were reported in pSS (88). Although hyporesponsiveness is not restricted to B cells, in the current study, T cells from AID patients did not show enhanced PTP/PSP activities, indicating a dysfunctional regulatory process restricted to B cells.

TLR9 activation facilitates the development of autoantibodies against dsDNA and ribonucleoproteins, indicating a critical role of this receptor in the activation of B cells in autoimmunity (89, 90). It activates memory B cells and drive *in vitro* proliferation and differentiation of B cells into ASCs (71, 72) and is considered to be involved in type I interferon production in autoimmunity (91). However, we found that AID B cells responded weakly to TLR9 activation *in vitro*. This is consistent with other studies describing reduced responses of SLE B cells against pokeweed mitogen (92); reduced IL-6, IL-10, vascular endothelial growth factor (VEGF), and IL-1ra production and reduced Ki-67 expression (43, 44); and reduced frequencies of CD69^+^CD86^+^ and TACI^+^CD25^+^ B cells after TLR9 *in vitro* stimulation (44). We and others (69, 70) found a functional connection between TLR9 and BCR signaling since Syk inhibition had an impact on TLR9 responses of normal B cells. This finding suggests that the abnormality in TLR9 signaling may be part of the anergic phenotype in SLE and reflect the reduced Syk signaling induced by repetitive BCR engagement *in vivo* in this disease.

Some reports indicate a role of reduced BCR signaling in the development and progression of autoimmunity (93, 94). Interestingly, inhibition of PTPN22 could reset central B cell tolerance in NOD *scid* gamma chain knock out (NSG) mice, which were engrafted with human hematopoietic stem cells carrying the gain of function mutation of *PTPN22* (94). Whereas, this study indicates that a normalized BCR signal could restore immune tolerance, the strong role of CD40 activation in *PTPN22* risk gene carriers is also consistent with the idea that this pathway is critical for censoring the overly active immune system in autoimmunity (95). Whether PTPN22 variant increases or decreases BCR signaling is a matter of debate. Mice expressing this mutation displayed enhanced BCR and CD40 responses (96). CD40 seems to be a critical context-dependent co-stimulatory molecule regulating both the full activation of BCR-stimulated B cells as well as their subsequent censoring. In this context, the current result that CD40 co-stimulation can render AID dysfunctional B cells susceptible to BCR stimulation and that CD40 treatment of SLE B cells diminished the expression of, e.g., PTPN22 further support that modulation of the CD40 pathway is of critical importance in regulating B cell function at many levels.

The effectiveness of B cell-directed therapies, such as rituximab (anti-CD20^+^) in RA, or belimumab (anti-BAFF/BLyS) in SLE, underscores the role of B cells in these diseases (97, 98). Our study suggests that blocking CD40/CD40L interaction by fostering B cell anergy holds promise to interfere with the cycle of B cell activation, tissue damage, and inflammation in AID. In fact, a study using an anti-CD154 antibody could prevent ASC generation in SLE patients (99). A second-generation PEGylated monoclonal antibody is currently in clinical development for SLE (100). Our data support that this treatment would be beneficial in RA and pSS as well.

In conclusion, we found that SLE B cells and RA and pSS memory B cells exhibit diminished responsiveness to BCR and TLR9 signaling, which may reflect a status of post-activation functional anergy owing to *in vivo* engagement of the BCR without appropriate T cell-derived co-stimulation. CD40 activation of B cells is critical to overcome this state of diminished responsiveness and restore BCR responses. Our findings support the investigation of new therapeutic options that interfere with the interaction between CD40/CD154 and, thereby, foster functional B cell anergy and decreased disease activity.

## Data Availability

The RNASeq data sets analyzed from the literature can be found in NCBI gene expression omnibus (GSE4588, CD20^+^ B cells; GSE117935, CD19^+^ B cells) and ArrayExpress platform (E-MTAB-2713, CD4^+^ and CD8^+^ T cells). The RNASeq data generated for this study are available in NCBI sequence read archive (PRJNA564980).

## Ethics Statement

This study was carried out in accordance with the recommendations of the ethics' committee at the Charité University Hospital Berlin with written informed consent from all subjects. All subjects gave written informed consent in accordance with the Declaration of Helsinki.

## Author Contributions

The concept of the study was developed by AL, PL, JW, LR, and TD. Data were obtained and analyzed by SW, AL, FS, AWi, HR-A, A-LS, PB, AWe, AJ, KN, JW, ES, GN, JI-K, MC, and AG. All authors developed, read, and approved the current manuscript.

### Conflict of Interest Statement

The authors declare that the research was conducted in the absence of any commercial or financial relationships that could be construed as a potential conflict of interest.
