# Supplementary material for: Identification and Characterization of Post-activated B Cells in Systemic Autoimmune Diseases
Source: Front Immunol. 2019 Sep 24;10:2136. doi: 10.3389/fimmu.2019.02136 (PMC6768969; doi:10.3389/fimmu.2019.02136)
Supplement: Supplementary file 1 [file Data_Sheet_1.PDF]

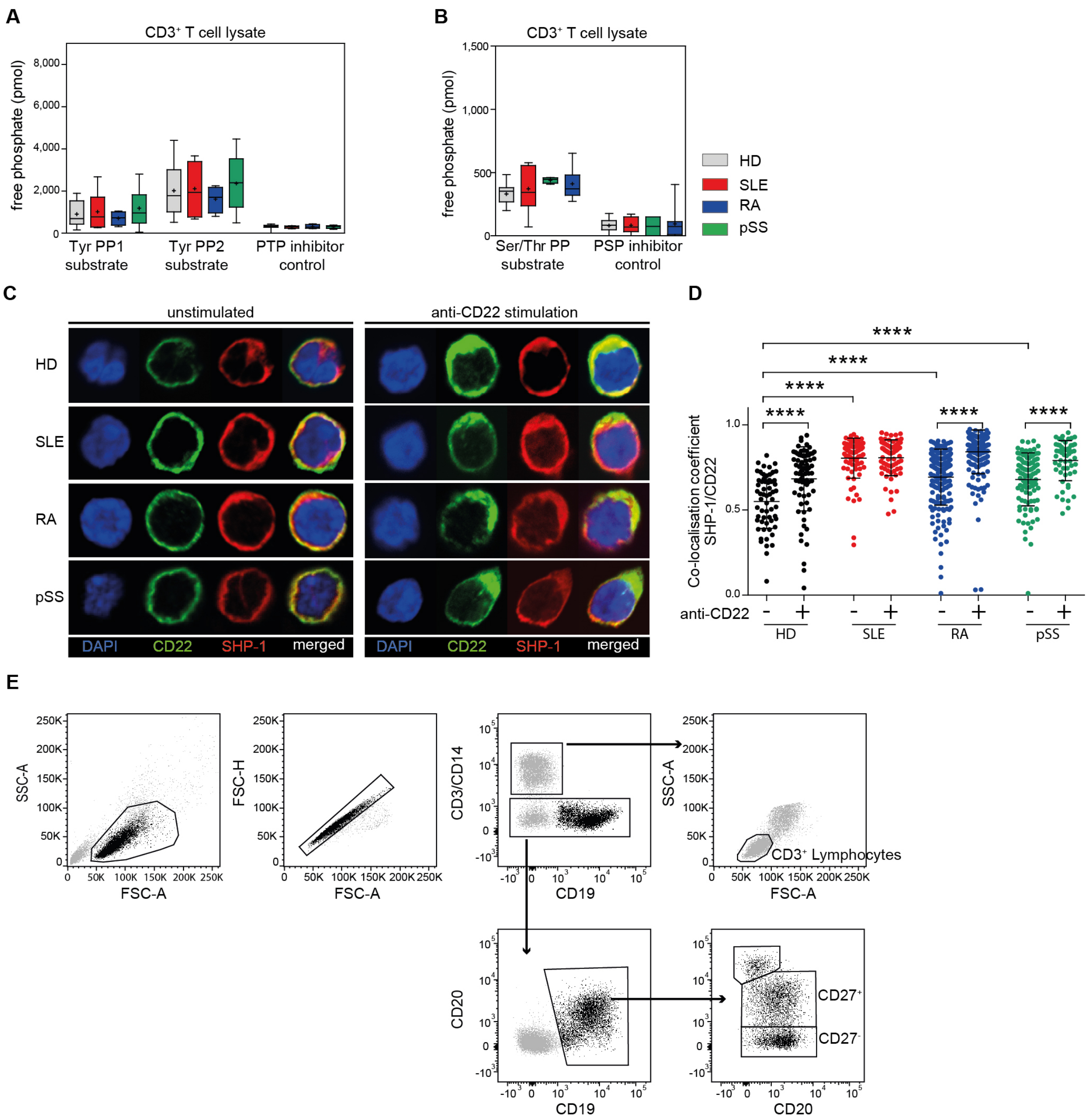

Figure S1. Similar phosphatase activities in T cells and enhanced baseline co-localization of CD22/SHP-1 in B cells from patients with AID. (**A**) PTP (n(HD/SLE/RA/pSS) = 14/10/4/10) and (**B**) PSP (n(HD/SLE/RA/pSS) = 13/9/4/9) activities from CD3^{+} T cells in HD (grey), SLE (red), RA (blue) and pSS (green). (**C**) Representative confocal microscopy pictures of CD22 (green) and SHP-1 (red) co-localization on HD, SLE, RA and pSS B cells. The nucleus (blue) was stained with DAPI. (**D**) Co-localization coefficients for HD (black), SLE (red), RA (blue) and pSS (green) B cells (n(HD/SLE/RA/pSS) = 2/2/2/2; were analyzed). Each dot represents a cell. (**E**) Gating strategy for the analysis of CD27^{-} B cells and CD27^{+} conventional memory B cells. CD3^{+} lymphocytes cells were identified within the CD3^{+}CD14^{+} cells by their scatter properties (unstimulated negative control). Box whisker plots represent median (line), mean (plus) and the range from minimum to maximum. The lines in the scatter dot plots indicate the mean ± SD (ANOVA with DMCT; **** $p < 0.0001$).
