# Supplementary material for: Identification and Characterization of Post-activated B Cells in Systemic Autoimmune Diseases
Source: Front Immunol. 2019 Sep 24;10:2136. doi: 10.3389/fimmu.2019.02136 (PMC6768969; doi:10.3389/fimmu.2019.02136)
Supplement: Supplementary file 2 [file Data_Sheet_2.PDF]

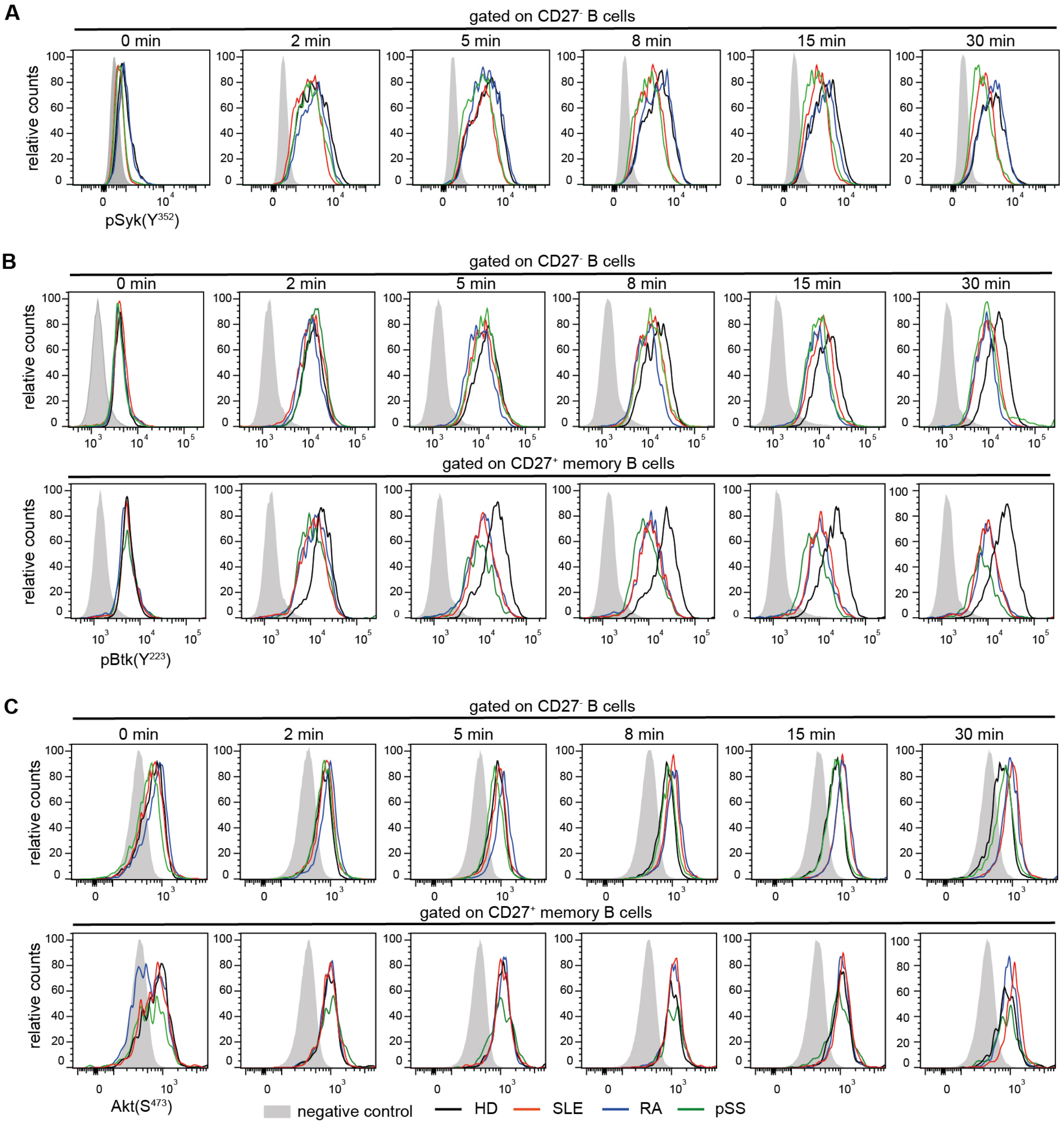

Figure S2: Reduced PTK, but not Akt serine phosphorylation upon BCR signaling in CD27^{+} B cells from AID. Representative histograms of CD27^{-} and CD27^{+} B cells stimulated for 0 (= unstimulated), 2, 5, 8, 15 and 30 min with anti-IgG/IgM. (**A**) pSyk(Y^{352}) in CD27^{-}, (**B**) pBtk(Y^{223}) in CD27^{-} and CD27^{+} and (**C**) pAkt(S^{473}) in CD27^{-} and CD27^{+} B cells from HD (black lines), SLE (red lines), RA (blue lines) and pSS (green lines) are shown. Grey areas indicate unstimulated CD3^{+} T cells as controls.
