# Supplementary material for: Identification and Characterization of Post-activated B Cells in Systemic Autoimmune Diseases
Source: Front Immunol. 2019 Sep 24;10:2136. doi: 10.3389/fimmu.2019.02136 (PMC6768969; doi:10.3389/fimmu.2019.02136)
Supplement: Supplementary file 3 [file Data_Sheet_3.PDF]

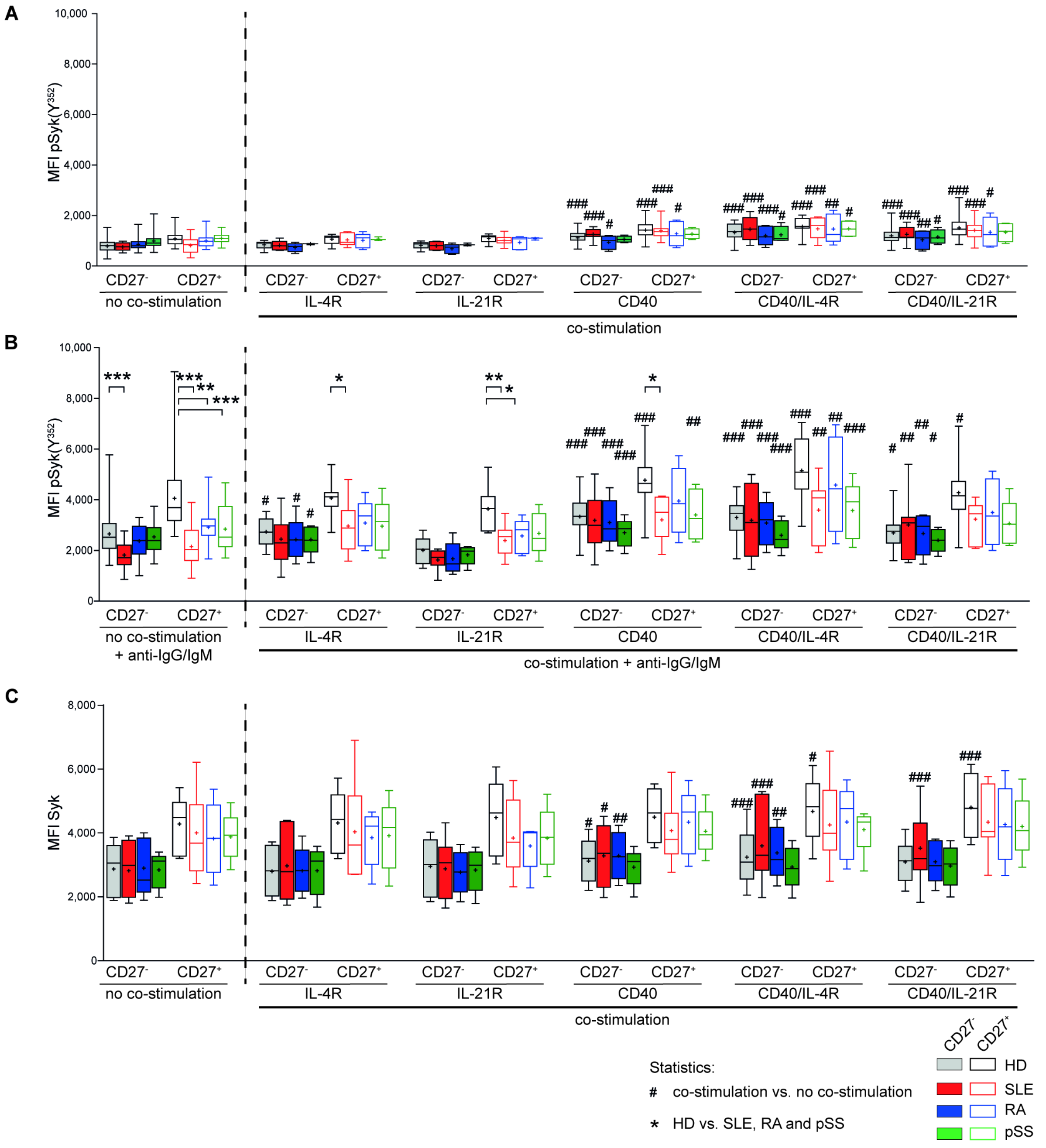

Figure S3: Enhanced Syk(Y^{352}) phosphorylation upon co-stimulation with combinations of CD40L, IL-4 and IL-21. PBMCs from HD (black), SLE (red), RA (blue) and pSS (green) patients were primed with IL-4, IL-21 and CD40L alone or in combination and subsequently stimulated with anti-IgG/IgM F(ab)$_2$. (**A**) Baseline and (**B**) anti-IgG/IgM induced pSyk(Y^{352}) in CD27$^-$ (filled boxes) and CD27$^+$ memory (blank boxes) B cells with (n(HD/SLE/RA/pSS) = 11/7/5/5) and without (n(HD/SLE/RA/pSS) = 30/18/16/15) indicated co-stimulation. (**C**) Syk expression upon co-stimulation. Box whisker plots represent median (line), mean (plus) and the range from minimum to maximum; lines in scatter dot plots represent means (ANOVA with DMCT, * $p \leq 0.05$, ** $p \leq 0.01$, *** $p \leq 0.001$; repeated measures ANOVA with DMCT, # $p \leq 0.05$, ## $p \leq 0.01$, ### $p \leq 0.001$).
