# Supplementary material for: Identification and Characterization of Post-activated B Cells in Systemic Autoimmune Diseases
Source: Front Immunol. 2019 Sep 24;10:2136. doi: 10.3389/fimmu.2019.02136 (PMC6768969; doi:10.3389/fimmu.2019.02136)
Supplement: Supplementary file 4 [file Data_Sheet_4.PDF]

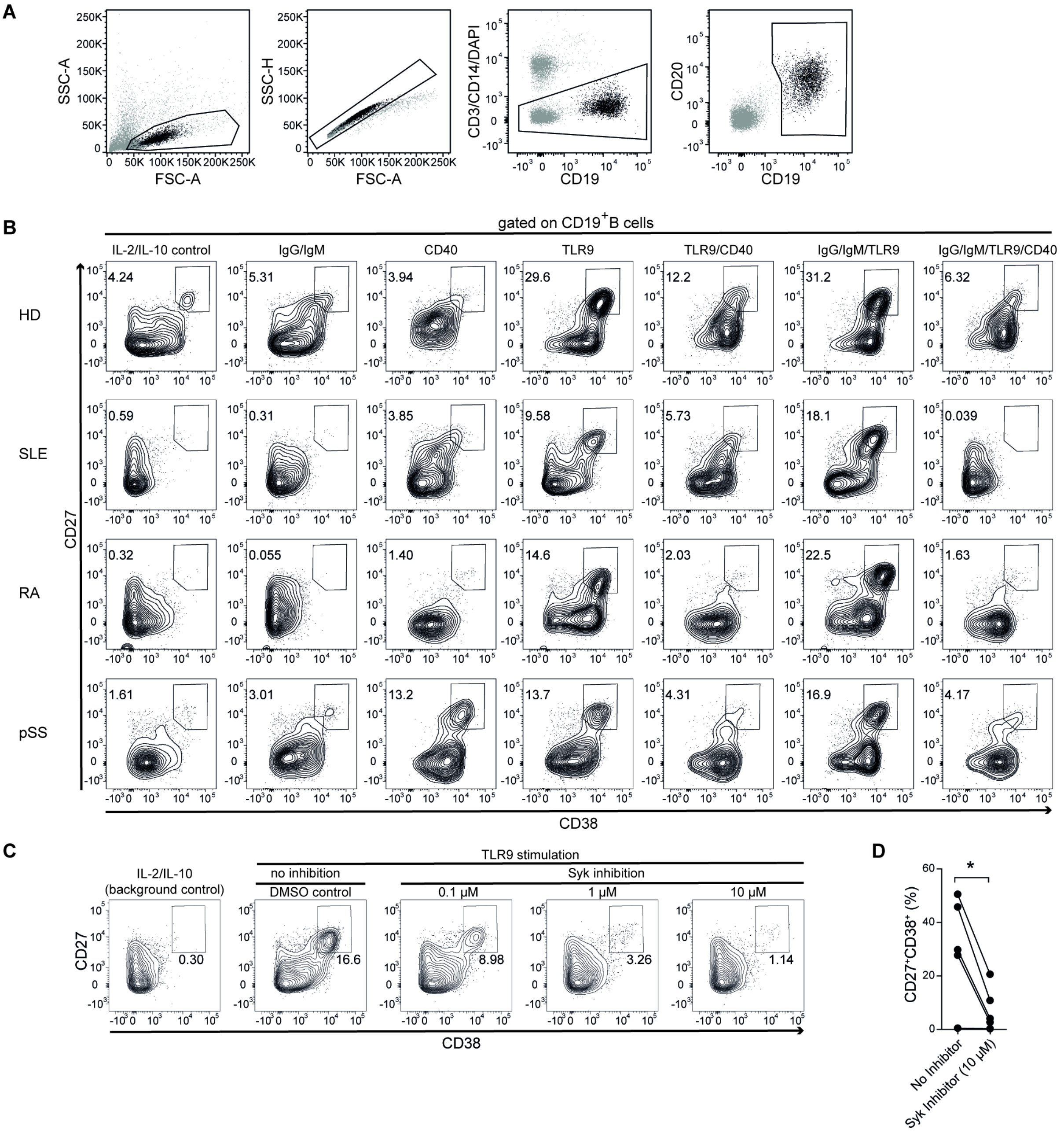

Figure S4: Reduced ASC differentiation of B cells from AID patients upon TLR9 stimulation. PBMCs from HD and AID patients were stimulated *in vitro* for 5 days. (**A**) Gating strategy for the analysis of $CD19^+$ B cells after culture. (**B**) Representative contour plots of $CD27^+CD38^+$ differentiated $CD19^+$ B cells from SLE, RA, pSS and HD (stimulation conditions are indicated). (**C**) Representative example of $CD27^+CD38^+$ B cells frequencies upon concentration dependent Syk inhibition including background and DMSO control. (**D**) $CD27^+CD38^+$ B cells frequencies of CpG stimulated cells with and without 10 µM Syk inhibitor (n(HD) = 5) (t-test; * $p \leq 0.05$).
