# Supplementary material for: Identification and Characterization of Post-activated B Cells in Systemic Autoimmune Diseases
Source: Front Immunol. 2019 Sep 24;10:2136. doi: 10.3389/fimmu.2019.02136 (PMC6768969; doi:10.3389/fimmu.2019.02136)
Supplement: Supplementary file 5 [file Data_Sheet_5.PDF]

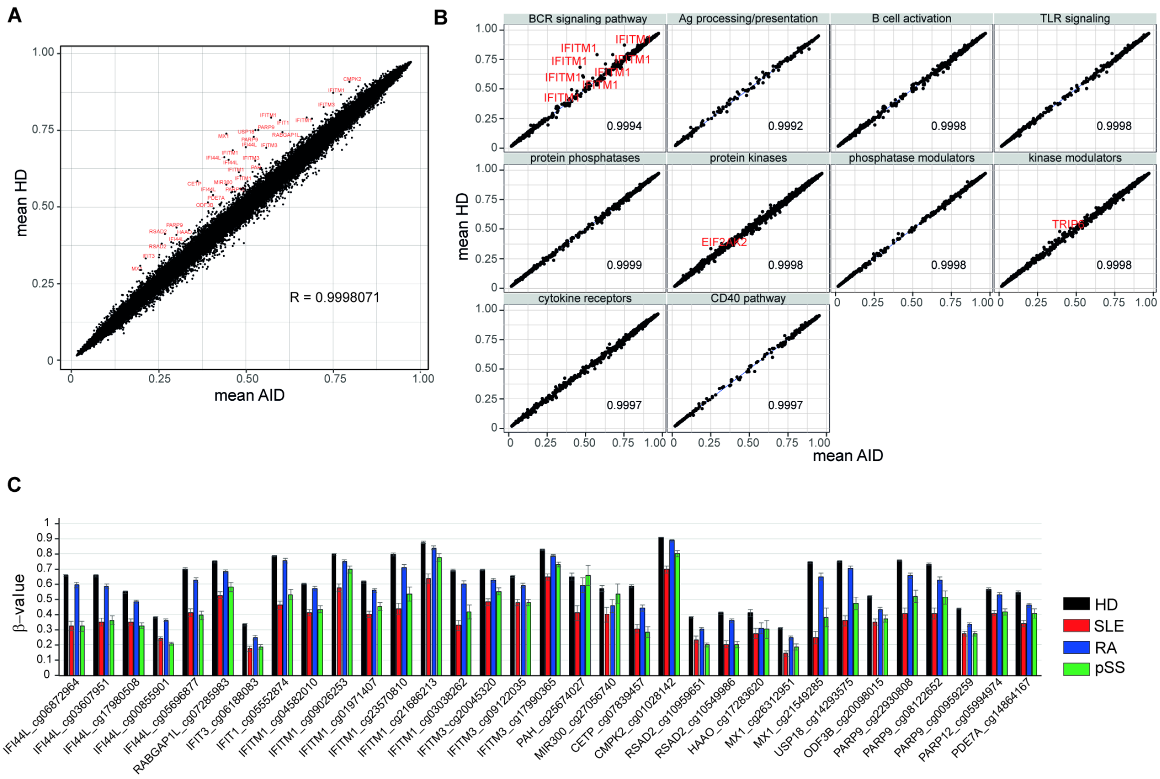

Figure S5: Similar methylation pattern of B cells from HD and AID patients. (**A**) Scatter plot representing the mean β-values of total single CpGs in HD and AID samples (n(HD/SLE/RA/pSS) = 175/48/49/24). (**B**) DNA methylation of CpGs associated with indicated categories was compared between HD and AID samples. (**C**) Individual CpGs that showed at least 10 % lower DNA methylation in AID compared to HD in (**A**) were analyzed in RA, pSS and SLE. Data shown are represented as mean ± SEM.
