# Supplementary material for: Identification and Characterization of Post-activated B Cells in Systemic Autoimmune Diseases
Source: Front Immunol. 2019 Sep 24;10:2136. doi: 10.3389/fimmu.2019.02136 (PMC6768969; doi:10.3389/fimmu.2019.02136)
Supplement: Supplementary file 6 [file Data_Sheet_6.PDF]

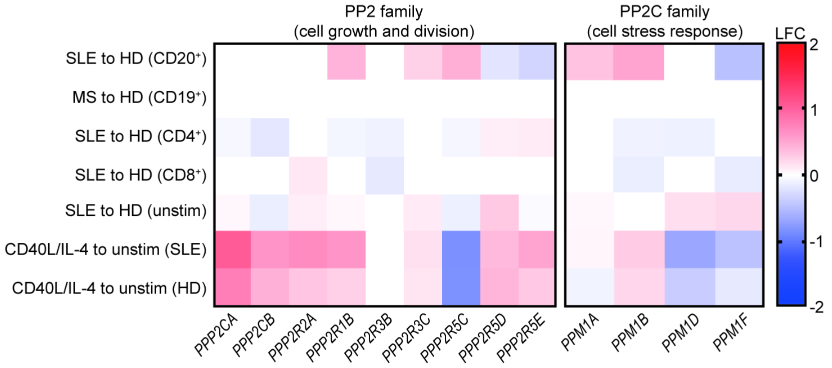

Figure S6: Common increase in PSP expression between HD and SLE after CD40L/IL-4 stimulation. Differential expression of selected genes related to PP2 and PP2C family PSPs in SLE vs. HD $CD20^+$ B cells (6 SLE, 7 HD), MS vs. HD $CD19^+$ B cells (10 MS, 10 HD), SLE vs. HD $CD4^+$ (53 SLE, 41 HD) and $CD8^+$ T cells (22 SLE, 31 HD) from the literature. Differential gene expression from un-stimulated SLE vs HD and CD40L/IL-4 stimulated for SLE versus unstimulated SLE or HD $CD19^+$ B cells, respectively (n(HD/SLE) = 1/2).
