# Supplementary material for: Identification and Characterization of Post-activated B Cells in Systemic Autoimmune Diseases
Source: Front Immunol. 2019 Sep 24;10:2136. doi: 10.3389/fimmu.2019.02136 (PMC6768969; doi:10.3389/fimmu.2019.02136)
Supplement: Supplementary file 7 [file Table_1.DOCX]

Supplementary Table 1: Donor information.

| **Donor No** | **Ethnicity** | **Gender** | **Age** | **Disease index**  SLE: SLEDAI  RA: DAS28  Sjögren’s: ESSDAI | **Medication** |
| --- | --- | --- | --- | --- | --- |
| HD1 | Caucasian | f | 27 | - | - |
| HD2 | Caucasian | f | 27 | - | - |
| HD3 | Caucasian | f | 26 | - | - |
| HD4 | Caucasian | f | 25 | - | - |
| HD5 | Caucasian | f | 26 | - | - |
| HD6 | Caucasian | f | 26 | - | - |
| HD7 | Caucasian | f | 21 | - | - |
| HD8 | Caucasian | f | 29 | - | - |
| HD9 | Caucasian | f | 28 | - | - |
| HD10 | Caucasian | m | 28 | - | - |
| HD11 | Caucasian | f | 65 | - | - |
| HD12 | Caucasian | m | 40 | - | - |
| HD13 | Caucasian | f | 33 | - | - |
| HD14 | Caucasian | f | 25 | - | - |
| HD15 | Caucasian | f | 22 | - | - |
| HD16 | Caucasian | f | 31 | - | - |
| HD17 | Caucasian | f | 24 | - | - |
| HD18 | Caucasian | f | 63 | - | - |
| HD19 | Caucasian | f | 27 | - | - |
| HD20 | Caucasian | f | 22 | - | - |
| HD21 | Caucasian | m | 29 | - | - |
| HD22 | Caucasian | f | 27 | - | - |
| HD23 | Caucasian | f | 27 | - | - |
| HD24 | Caucasian | f | 23 | - | - |
| HD25 | Caucasian | m | 36 | - | - |
| HD26 | Caucasian | m | 33 | - | - |
| HD27 | Caucasian | m | 49 | - | - |
| HD28 | Caucasian | m | 28 | - | - |
| HD29 | Caucasian | f | 40 | - | - |
| HD30 | Caucasian | m | 23 | - | - |
| HD31 | Caucasian | m | 23 | - | - |
| HD32 | Caucasian | m | 30 | - | - |
| HD33 | Caucasian | f | 26 | - | - |
| HD34 | Caucasian | f | 32 | - | - |
| HD35 | Caucasian | f | 50 | - | - |
| HD36 | Caucasian | f | 33 | - | - |
| HD37 | Caucasian | f | 27 | - | - |
| HD38 | Caucasian | f | 23 | - | - |
| HD39 | Caucasian | f | 32 | - | - |
| HD40 | Caucasian | f | 28 | - | - |
| HD41 | Caucasian | f | 23 | - | - |
| HD42 | Caucasian | f | 27 | - | - |
| HD43 | Caucasian | f | 30 | - | - |
| HD44 | Caucasian | f | 38 | - | - |
| HD45 | Caucasian | f | 28 | - | - |
| HD46 | Caucasian | f | 34 | - | - |
| HD47 | Caucasian | f | 35 | - | - |
| HD48 | Caucasian | f | 38 | - | - |
| HD49 | Caucasian | f | 23 | - | - |
| HD50 | Caucasian | f | 46 | - | - |
| HD51 | Caucasian | m | 55 | - | - |
| HD52 | Caucasian | m | 35 | - | - |
| HD53 | Caucasian | f | 31 | - | - |
| HD54 | Caucasian | f | 31 | - | - |
| HD55 | Caucasian | f | 29 | - | - |
| HD56 | Caucasian | f | 59 | - | - |
| HD57 | Caucasian | f | 25 | - | - |
| HD58 | Caucasian | m | 25 | - | - |
| HD59 | Caucasian | f | 25 | - | - |
| HD60 | Caucasian | f | 32 | - | - |
| HD61 | Caucasian | f | 39 | - | - |
| HD62 | Caucasian | f | 40 | - | - |
| HD63 | Caucasian | f | 60 | - | - |
| HD64 | Caucasian | f | 43 | - | - |
| HD65 | Caucasian | f | 30 | - | - |
| HD66 | Caucasian | f | 25 | - | - |
| HD67 | Caucasian | f | 24 | - | - |
| HD68 | Caucasian | f | 44 | - | - |
| HD69 | Caucasian | f | 39 | - | - |
| HD70 | Indian | f | 27 | - | - |
| HD71 | Caucasian | m | 36 | - | - |
| HD72 | Caucasian | m | 23 | - | - |
| HD73 | Caucasian | m | 30 | - | - |
| HD74 | Caucasian | m | 34 | - | - |
| HD75 | Caucasian | f | 28 | - | - |
| HD76 | Caucasian | f | 28 | - | - |
| HD77 | Caucasian | f | 30 | - | - |
| HD78 | Caucasian | f | 30 | - | - |
| HD79 | Caucasian | f | 23 | - | - |
| HD80 | Caucasian | f | 34 | - | - |
| HD81 | Caucasian | f | 35 | - | - |
| HD82 | Caucasian | f | 38 | - | - |
| HD83 | Caucasian | f | 49 | - | - |
| HD84 | Caucasian | f | 24 | - | - |
| HD85 | Caucasian | m | 24 | - | - |
| HD86 | Caucasian | f | 24 | - | - |
| HD87 | Caucasian | f | 29 | - | - |
| HD88 | Caucasian | m | 26 | - | - |
| HD89 | Caucasian | f | 23 | - | - |
| HD90 | Caucasian | m | 23 | - | - |
| HD91 | Caucasian | f | 24 | - | - |
| HD92 | Caucasian | f | 43 | - | - |
| HD93 | Caucasian | m | 35 | - | - |
| HD94 | Caucasian | f | 24 | - | - |
| HD95 | Caucasian | m | 37 | - | - |
| HD96 | Caucasian | f | 31 | - | - |
| HD97 | Caucasian | f | 28 | - | - |
| HD98 | Caucasian | f | 39 | - | - |
| HD99 | Caucasian | f | 30 | - | - |
| HD100 | Caucasian | f | 58 | - | - |
| HD101 | Caucasian | f | 30 | - | - |
| HD102 | Caucasian | f | 30 | - | - |
| HD103 | Caucasian | f | 39 | - | - |
| HD104 | Caucasian | f | 23 | - | - |
| HD105 | Caucasian | m | 53 | - | - |
| HD106 | Caucasian | f | 57 | - | - |
| HD107 | Caucasian | f | 58 | - | - |
| HD108 | Caucasian | f | 30 | - | - |
| HD109 | Caucasian | f | 31 | - | - |
| HD110 | Caucasian | f | 40 | - | - |
| HD111 | Caucasian | f | 31 | - | - |
| HD112 | Caucasian | f | 33 | - | - |
| HD113 | Caucasian | f | 37 | - | - |
| HD114 | Caucasian | f | 41 | - | - |
| HD115 | Caucasian | f | 29 | - | - |
| HD116 | Caucasian | f | 30 | - | - |
| HD117 | Caucasian | f | 55 | - | - |
| HD118 | Caucasian | m | 25 | - | - |
| SLE1 | Caucasian | f | 30 | 10.00 | MTX, Pred, HCQ |
| SLE2 | Caucasian | f | 32 | <6 | Pred |
| SLE3 | Asian | f | 20 | 6.00 | Pred, HCQ |
| SLE4 | Caucasian | f | 60 | <6 | MTX |
| SLE5 | Caucasian | f | 61 | <6 | Pred, AZA |
| SLE6 | Caucasian | f | 39 | 10.00 | Pred, AZA, HCQ |
| SLE7 | Caucasian | f | 33 | 5.00 | Pred |
| SLE8 | Caucasian | f | 38 | <6 | none |
| SLE9 | Caucasian | f | 32 | 13.00 | Pred, HCQ |
| SLE10 | Caucasian | f | 21 | 27.00 | Pred |
| SLE11 | Caucasian | f | 54 | 10.00 | Pred, HCQ |
| SLE12 | Caucasian | f | 40 | <6 | Pred, AZA |
| SLE13 | Caucasian | f | 31 | <6 | Pred, AZA |
| SLE14 | Caucasian | f | 28 | <6 | Pred |
| SLE15 | Caucasian | f | 62 | <6 | Pred, MMF |
| SLE16 | Caucasian | f | 31 | 7.00 | none |
| SLE17 | Caucasian | f | 73 | <6 | none |
| SLE18 | Caucasian | f | 35 | <6 | Pred |
| SLE19 | Caucasian | f | 28 | <6 | Pred |
| SLE20 | Caucasian | f | 53 | ~6 | Pred, HCQ |
| SLE21 | Caucasian | f | 39 | 10.00 | AZA, HCQ |
| SLE22 | Caucasian | f | 28 | 2.00 | MTX, HCQ |
| SLE23 | Caucasian | f | 42 | 4.00 | HCQ |
| SLE24 | Caucasian | f | 30 | <6 | Pred |
| SLE25 | Caucasian | f | 35 | 12.00 | Pred, AZA |
| SLE26 | Caucasian | f | 28 | <6 | Pred |
| SLE27 | Caucasian | f | 61 | <6 | MTX |
| SLE28 | Caucasian | f | 29 | <6 | Pred, HCQ, Belimumab |
| SLE29 | Caucasian | f | 31 | 4.00 | HCQ |
| SLE30 | Caucasian | f | 54 | 8.00 | Pred, HCQ |
| SLE31 | Caucasian | f | 41 | <6 | Pred, HCQ |
| SLE32 | Caucasian | f | 45 | <6 | Pred, HCQ |
| SLE33 | Caucasian | f | 32 | 7.00 | HCQ |
| SLE34 | Caucasian | m | 44 | 15.00 | Pred, HCQ |
| SLE35 | Caucasian | f | 49 | <6 | Pred, HCQ |
| SLE36 | Caucasian | f | 62 | <6 | MTX |
| SLE37 | Caucasian | f | 41 | <6 | Pred, AZA |
| SLE38 | Caucasian | f | 19 | <6 | HCQ, MMF |
| SLE39 | Caucasian | f | 35 | 3.00 | HCQ, MMF, Belimumab |
| SLE40 | Caucasian | f | 31 | 2.00 | Pred, AZA, HCQ |
| SLE41 | Caucasian | f | 32 | <6 | HCQ |
| SLE42 | Caucasian | f | 55 | <6 | AZA, HCQ |
| SLE43 | African | f | 38 | 18.00 | Pred, MMF, HCQ |
| SLE44 | Caucasian | m | 25 | <6 | AZA |
| SLE45 | Caucasian | f | 30 | 7.00 | none |
| SLE46 | Caucasian | f | 30 | 6 | MTX |
| SLE47 | Caucasian | f | 28 | 3.00 | HCQ |
| SLE48 | Caucasian | f | 34 | 4.00 | HCQ |
| SLE49 | Caucasian | w | 56 | 4.00 | Pred, HCQ |
| SLE50 | Caucasian | m | 23 | 4.00 | Pred, HCQ, MMF, Belimumab |
| SLE51 | Caucasian | f | 38 | 4.00 | Pred, AZA, HCQ |
| SLE52 | Caucasian | f | 37 | <6 | Pred, HCQ, Cyclo |
| SLE53 | Caucasian | f | 30 | <6 | Pred, AZA |
| SLE54 | Caucasian | f | 30 | 6.00 | HCQ |
| SLE55 | Caucasian | f | 26 | 8.00 | Pred, HCQ, Cyclo |
| SLE56 | Caucasian | f | 62 | <6 | Pred, Cyclo |
| SLE57 | Caucasian | f | 25 | 2.00 | Cyclo |
| SLE58 | Caucasian | m | 64 | 4.00 | Pred, HCQ, MMF |
| SLE59 | Caucasian | f | 73 | 0.00 | none |
| SLE60 | Caucasian | f | 41 | 2.00 | MTX, Pred, HCQ |
| SLE61 | Caucasian | f | 45 | 2.00 | Pred, HCQ |
| SLE62 | Caucasian | f | 62 | <6 | MTX |
| SLE63 | Caucasian | m | 52 | <6 | Pred, MMF |
| SLE64 | Caucasian | f | 56 | <6 | HCQ |
| SLE65 | Caucasian | f | 45 | <6 | Pred |
| SLE66 | Caucasian | f | 41 | 6.00 | Pred, AZA |
| SLE67 | Caucasian | f | 30 | <6 | Pred, AZA |
| SLE68 | Caucasian | f | 53 | 7.00 | HCQ, MTX, Pred |
| SLE69 | Caucasian | m | 22 | 12.00 | MMF, Pred |
| SLE70 | Caucasian | f | 36 | 4.00 | Pred, HCQ, Cyclo |
| SLE71 | Caucasian | f | 35 | 9.00 | AZA, Pred |
| SLE72 | Caucasian | f | 45 | 21.00 | none |
| SLE73 | Caucasian | f | 27 | 4.00 | Pred |
| SLE74 | Caucasian | f | 30 | 8.00 | CyA, Pred |
| SLE75 | Caucasian | m | 48 | <6 | Pred, Cyclo |
| SLE76 | Caucasian | f | 28 | 10.00 | AZA, Pred |
| SLE77 | Caucasian | f | 39 | 8.00 | AZA, Pred |
| SLE78 | Caucasian | f | 29 | 14.00 | Pred, HCQ |
| SLE79 | Caucasian | f | 34 | 5.00 | MMF, HCQ, Pred |
| SLE80 | Caucasian | f | 64 | 3.00 | none |
| SLE81 | Caucasian | f | 46 | 6.00 | MMF, Pred |
| SLE82 | Caucasian | f | 54 | 4.00 | HCQ, Pred |
| SLE83 | Caucasian | f | 30 | 6.00 | CyA, Pred |
| SLE84 | Caucasian | f | 32 | 4.00 | Pred |
| SLE85 | Caucasian | f | 27 | 4.00 | Pred |
| RA1 | Caucasian | f | 79 | 2.85 | Pred, Toci |
| RA2 | Caucasian | m | 59 | < 3.2 | MTX, Pred |
| RA3 | Caucasian | f | 52 | 3.2 - 5.1 | MTX, Pred |
| RA4 | Caucasian | f | 61 | <2.6 | Pred, Toci |
| RA5 | Caucasian | m | 56 | 3.2 - 5.1 | MTX, Pred, Sulfa |
| RA6 | Caucasian | m | 39 | 4.97 | MTX, Pred, Etanercept |
| RA7 | Caucasian | f | 58 | <3.2 | MTX, Pred, Abatacept |
| RA8 | Caucasian | f | 26 | <2.6 | none |
| RA9 | Caucasian | f | 51 | 4.86 | Pred |
| RA10 | Caucasian | f | 58 | 5.74 | Pred |
| RA11 | Caucasian | f | 54 | nb | MTX |
| RA12 | Caucasian | f | 69 | 3.2 - 5.1 | Pred, Toci |
| RA13 | Caucasian | f | 33 | <2.6 | Pred |
| RA14 | Caucasian | f | 50 | <2.6 | MTX |
| RA15 | Caucasian | f | 29 | <2.6 | Pred |
| RA16 | Caucasian | f | 25 | <3.2 | MTX |
| RA17 | Caucasian | m | 57 | 2.85 | Pred, Toci, Sulfa |
| RA18 | Caucasian | f | 64 | <2.6 | Etanercept |
| RA19 | Caucasian | m | 60 | <3.2 | MTX, Pred |
| RA20 | Caucasian | f | 57 | <3.2 | MTX, Pred |
| RA21 | Caucasian | f | 29 | <3.2 | Pred |
| RA22 | Caucasian | f | 54 | 5.20 | MTX, Pred |
| RA23 | Caucasian | f | 45 | < 3,2 | MTX |
| RA24 | Caucasian | f | 46 | <3.2 | MTX |
| RA25 | Caucasian | f | 66 | 1.53 | Toci |
| RA26 | Caucasian | m | 71 | 2.50 | MTX, Simponi |
| RA27 | Caucasian | f | 65 | 1.75 | Pred, Toci |
| RA28 | Caucasian | f | 78 | 3.85 | Pred, Toci |
| RA29 | Caucasian | m | 55 | 7.30 | Pred, Enbrel |
| RA30 | Caucasian | f | 55 | 6.50 | MTX, Abatacept |
| RA31 | Caucasian | f | 48 | 2.60 | MTX, Pred |
| RA32 | Caucasian | f | 68 | 5.20 | Pred, Enbrel |
| RA33 | Caucasian | f | 26 | <3,2 | MTX, Enbrel |
| RA34 | Caucasian | f | 77 | 3.60 | MTX, Pred, Enbrel |
| RA35 | Caucasian | m | 61 | 5.30 | Etanercept |
| RA36 | Caucasian | m | 58 | 1.36 | Pred |
| RA37 | Caucasian | f | 54 | 5.20 | MTX, Pred |
| RA38 | Caucasian | f | 45 | 1.36 | MTX, Etanercept |
| RA39 | Caucasian | f | 74 | 2.68 | MTX |
| RA40 | Caucasian | f | 74 | 5.39 | MTX, Pred |
| RA41 | Caucasian | f | 88 | 2.70 | MTX, Pred |
| RA42 | Caucasian | f | 60 | 2.80 | MTX, Enbrel |
| pSS1 | Caucasian | f | 56 | 0 | none |
| pSS2 | Caucasian | f | 78 | 3 | CYC |
| pSS3 | Caucasian | f | 58 | 2 | Pred |
| pSS4 | Caucasian | f | 57 | 2 | none |
| pSS5 | Caucasian | f | 79 | 1 | Pred |
| pSS6 | Caucasian | f | 63 | 0 | none |
| pSS7 | Caucasian | f | 52 | 3 | Pred, AZA |
| pSS8 | Caucasian | f | 62 | 2 | none |
| pSS9 | Caucasian | m | 37 | 0 | none |
| pSS10 | Caucasian | f | 44 | 0 | Pred |
| pSS11 | Caucasian | f | 62 | 1 | HCQ |
| pSS12 | Caucasian | f | 63 | 0 | Pred |
| pSS13 | Caucasian | f | 80 | 2 | none |
| pSS14 | Caucasian | f | 44 | 6 | none |
| pSS15 | Caucasian | f | 51 | 0 | Pred |
| pSS16 | Caucasian | f | 62 | 2 | none |
| pSS17 | Caucasian | f | 76 | 1 | HCQ |
| pSS18 | Caucasian | f | 34 | 0 | Pred |
| pSS19 | Caucasian | f | 67 | 0 | Pred, HCQ |
| pSS20 | Caucasian | f | 45 | 0 | none |
| pSS21 | Caucasian | f | 61 | 5 | none |
| pSS22 | Caucasian | f | 79 | 0 | HCQ |
| pSS23 | Caucasian | f | 65 | 0 | HCQ |
| pSS24 | Caucasian | f | 40 | 0 | HCQ |
| pSS25 | Caucasian | f | 53 | 6 | HCQ |
| pSS26 | Caucasian | f | 39 | <4 | none |
| pSS27 | Caucasian | f | 78 | 5 | none |
| pSS28 | Caucasian | f | 34 | 5 | HCQ |
| pSS29 | Caucasian | f | 67 | <4 | Pred, HCQ |
| pSS30 | Caucasian | f | 67 | 6 | none |
| pSS31 | Caucasian | f | 56 | 0 | none |
| pSS32 | Caucasian | f | 30 | 1 | AZA, HCQ |
| pSS33 | Caucasian | f | 37 | 0 | HCQ |
| pSS34 | Caucasian | f | 30 | 2 | HCQ |
| pSS35 | Caucasian | f | 27 | 0 | HCQ |
| pSS36 | Caucasian | f | 60 | 1 | HCQ |
| pSS37 | Caucasian | f | 75 | <6 | none |
| pSS38 | Caucasian | f | 76 | 0 | HCQ |
| pSS39 | Caucasian | f | 78 | 0 | HCQ |
| pSS40 | Caucasian | f | 49 | 0 | Pred, HCQ |
| pSS41 | Caucasian | f | 68 | 0 | Pred, HCQ |
| pSS42 | Caucasian | f | 73 | 0 | none |
| pSS43 | Caucasian | f | 45 | 0 | CyA |
| pSS44 | Caucasian | f | 53 | 2 | none |
| pSS45 | Caucasian | f | 37 | 0 | HCQ |
| pSS46 | Caucasian | f | 36 | 0 | HCQ |
| pSS47 | Caucasian | f | 56 | 0 | HCQ |
| pSS48 | Caucasian | f | 78 | 3 | Pred, HCQ |
| pSS49 | Caucasian | f | 58 | 2 | none |
| pSS50 | Caucasian | f | 57 | 2 | HCQ |
| pSS51 | Caucasian | f | 79 | 1 | Pred, HCQ |

Annotations: Azathioprine (AZA), cyclophosphamide (Cyclo), hydroxychloroquine (HCQ), methotrexate (MTX), mycophenolatmofetil (MMF)*,* prednisolone (Pred), sulfasalazine (Sulfa), tocilizumab (Toci), Systemic lupus erythematosus disease activity index (SLEDAI), disease activity score-28 (DAS28), European league against rheumatism (EULAR) Sjögren’s syndrome disease activity index (ESSDAI).
