# Supplementary material for: Identification and Characterization of Post-activated B Cells in Systemic Autoimmune Diseases
Source: Front Immunol. 2019 Sep 24;10:2136. doi: 10.3389/fimmu.2019.02136 (PMC6768969; doi:10.3389/fimmu.2019.02136)
Supplement: Supplementary file 8 [file Table_2.DOCX]

Supplementary Table 2: List of stimulation and staining antibodies for flow cytometry analysis and confocal microscopy.

| target | conjugate | species | reactivity | clone | catalog number | company |
| --- | --- | --- | --- | --- | --- | --- |
| CD3 | PB | mouse IgG1 kappa | anti-human | UCHT1 | 558117 | BD Bioscience |
| CD4 | PerCP | mouse IgG1 kappa | anti-human | SK3 | 344624 | BioLegend |
| CD14 | APC-Cy7 | mouse IgG2a kappa | anti-human | M5E2 | 301820 | BioLegend |
| CD14 | PB | mouse IgG2a kappa | anti-human | M5E2 | 558121 | BD Bioscience |
| CD14 | AF488 | mouse IgG2a kappa | anti-human | 301811 | M5E2 | BioLegend |
| CD16 | APC-Cy7 | mouse IgG1 kappa | anti-human | 302018 | 3G8 | BioLegend |
| CD19 | PE-Cy7 | mouse IgG1 kappa | anti-human | SJ25C1 | 557835 | BD Bioscience |
| CD20 | BV510 | mouse IgG2b kappa | anti-human | 2H7 | 302340 | BioLegend |
| CD20 | PO | mouse IgG3 | anti-human | HI47 | MHCD2030 | Invitrogene |
| CD20 | PerCp-Cy5.5 | mouse IgG2a kappa | anti-human | H1(FB1) | 558021 | BD Bioscience |
| CD22 | PE | mouse IgG1 kappa | anti-human | S-HCL-1 | 555424 | BD Bioscience |
| CD27 | APC | mouse IgG1 kappa | anti-human | L128 | 337169 | BD Bioscience |
| CD38 | APC-Cy7 | mouse IgG1 kappa | anti-human | HIT2 | 303534 | BioLegend |
| Akt1 | PerCp-Vio700 | recombinant human IgG1 | anti-human | REA134 | 130-100-741 | Miltenyi Biotec GmbH |
| pAkt(S^473^) | PE | mouse IgG1 kappa | anti-human | M89-61 | 560378 | BD Bioscience |
| Biotin | APC | mouse IgG1 | - | Bio3-18E7 | 130-090-856 | Miltenyi Biotec GmbH |
| Btk | PE | mouse IgG2a | anti-human | 53/BTK | 558527 | BD Bioscience |
| pBtk(Y^223^) | FITC | mouse IgG1 kappa | anti-human | N35-86 | 564847 | BD Bioscience |
| SHP-1 | - | mouse IgG1 | Anti-human | Clone 52 | 610126 | BD Bioscience |
| Syk | FITC | mouse IgG2 a | anti-human | 4D10 | 552476 | BD Bioscience |
| pSyk(Y^352^) | PE | mouse IgG1 kappa | anti-human | 17A/P-ZAP70 | 557881 | BD Bioscience |
| PLCγ2 | PE | mouse IgG1 kappa | anti-human | K86-1161 | 560134 | BD Bioscience |
| pPLCγ2(Y^759^) | AF488 | mouse IgG1 kappa | anti-human | K86-689.37 | 558507 | BD Bioscience |
| IgG/IgM | - | goat F(ab’)_2_ | anti-human | - | **109-006-127** | Jackson ImmunoResearch |
| donkey-anti-mouse | RRX | donkey | anti-mouse | poly | **715-296-150** | Jackson ImmunoResearch |
| donkey-anti-goat | A647 | donkey | anti-goat | poly | **A-21447** | Invitrogen |
| CD22 F(ab’)2 (epratuzumab) | AF488 | humanized | anti-human | - | **-** | UCB Pharma |

Annotations: Pacific blue (PB), peridinin-chlorophyll-protein (PerCP), allophycocyanin (APC), phycoerythrin (PE), brilliant violet (BV), pacific orange (PO), fluorescein isothiocyanate (FITC), alexa flour (AF), cyanine (Cy), rhodamine red-X (RRX), protein kinase B (Akt1), Bruton’s tyrosine kinase (Btk), spleen tyrosine kinase (Syk), 1-Phosphatidylinositol-4,5-bisphosphate phosphodiesterase gamma-2 (PLCγ2),  Src homology region 2 domain-containing phosphatase-1 (SHP-1).
