# Supplementary material for: Identification and Characterization of Post-activated B Cells in Systemic Autoimmune Diseases
Source: Front Immunol. 2019 Sep 24;10:2136. doi: 10.3389/fimmu.2019.02136 (PMC6768969; doi:10.3389/fimmu.2019.02136)
Supplement: Supplementary file 9 [file Table_3.DOCX]

Supplementary Table 3: List of stimulation reagents and inhibitors.

| Target | reagent | catalog number | company |
| --- | --- | --- | --- |
| CD40 receptor | human CD40-Ligand  multimer kit | 130-098-775 | Miltenyi Biotec GmbH |
| IL-2 receptor | recombinant human IL-2 | 130-097-745 | Miltenyi Biotec GmbH |
| IL-4 receptor | recombinant human IL-4 | 200-04 | PeproTech |
| IL-10 receptor | recombinant human IL-10 | 130-093-948 | Miltenyi Biotec GmbH |
| IL-21 receptor | recombinant human IL-21 | 200-21 | PeproTech |
| PSP inhibitor | sodium fluoride | V2460 | Sigma-Aldrich Chemie |
| PTP inhibitor | sodium orthovanadat | V2471 | Sigma-Aldrich Chemie |
| protease inhibitor | halt protease inhibitor cocktail | 87786 | Thermo Fisher Scientific |
| Syk | entospletinib (GS-9973) | S7523 | Selleck Chemicals |
| TLR9 | CpG (ODN2006) | 130-100-106 | Miltenyi Biotec GmbH |

Annotations: Toll like receptor 9 (TLR9), spleen tyrosine kinase (Syk), protein serine/threonine phosphatase (PSP), protein tyrosine phosphatase (PTP), protein serine/threonine phosphatase (PSP).
