# Supplementary material for: Identification and Characterization of Post-activated B Cells in Systemic Autoimmune Diseases
Source: Front Immunol. 2019 Sep 24;10:2136. doi: 10.3389/fimmu.2019.02136 (PMC6768969; doi:10.3389/fimmu.2019.02136)
Supplement: Supplementary file 10 [file Table_4.DOCX]

Supplementary Table 4: List of differentially methylated CpG sides.

| Cg ID | gene ID | region annotation | chromosome | start | stop |
| --- | --- | --- | --- | --- | --- |
| cg06872964 | IFI44L | TSS1500 | chr1 | 79085250 | 79085251 |
| cg03607951 | IFI44L | TSS1500 | chr1 | 79085586 | 79085587 |
| cg17980508 | IFI44L | TSS1500 | chr1 | 79085713 | 79085714 |
| cg00855901 | IFI44L | TSS1500 | chr1 | 79085765 | 79085766 |
| cg05696877 | IFI44L | 5'UTR | chr1 | 79088769 | 79088770 |
| cg07285983 | RABGAP1L | body; TSS200 | chr1 | 174844490 | 174844491 |
| cg06188083 | IFIT3 | body; body | chr10 | 91093005 | 91093006 |
| cg05552874 | IFIT1 | body | chr10 | 91153143 | 91153144 |
| cg04582010 | IFITM1 | TSS1500 | chr11 | 313120 | 313121 |
| cg09026253 | IFITM1 | TSS1500 | chr11 | 313267 | 313268 |
| cg01971407 | IFITM1 | TSS1500 | chr11 | 313624 | 313625 |
| cg23570810 | IFITM1 | body | chr11 | 315102 | 315103 |
| cg21686213 | IFITM1 | 3'UTR | chr11 | 315118 | 315119 |
| cg03038262 | IFITM1 | 3'UTR | chr11 | 315262 | 315263 |
| cg20045320 | IFITM3 | downstream | chr11 | 319555 | 319556 |
| cg09122035 | IFITM3 | downstream | chr11 | 319667 | 319668 |
| cg17990365 | IFITM3 | 2^nd^ exon | chr11 | 319718 | 319719 |
| cg25674027 | PAH | upstream | chr12 | 103325781 | 103325782 |
| cg27056740 | MIR300 | body | chr14 | 101507727 | 101507728 |
| cg07839457 | CETP | TSS1500 | chr16 | 57023022 | 57023023 |
| cg01028142 | CMPK2 | body | chr2 | 7004578 | 7004579 |
| cg10959651 | RSAD2 | 1^st^ exon | chr2 | 7018020 | 7018021 |
| cg10549986 | RSAD2 | 1^st^ exon | chr2 | 7018153 | 7018154 |
| cg17283620 | HAAO | body | chr2 | 43013772 | 43013773 |
| cg26312951 | MX1 | TSS200; 5'UTR | chr21 | 42797847 | 42797848 |
| cg21549285 | MX1 | 5'UTR | chr21 | 42799141 | 42799142 |
| cg14293575 | USP18 | 5'UTR | chr22 | 18635460 | 18635461 |
| cg20098015 | ODF3B | TSS200 | chr22 | 50971140 | 50971141 |
| cg22930808 | PARP9 | 5'UTR | chr3 | 122281881 | 122281882 |
| cg08122652 | PARP9 | 5'UTR | chr3 | 122281939 | 122281940 |
| cg00959259 | PARP9 | 5'UTR | chr3 | 122281975 | 122281976 |
| cg05994974 | PARP12 | body | chr7 | 139761087 | 139761088 |
| cg14864167 | PDE7A | body | chr8 | 66751182 | 66751183 |

Annotations: Interferon-induced protein 44-like (IFI44L), Rab GTPase-activating protein 1-like (RABGAP1L), interferon-induced protein with tetratricopeptide repeats (IFIT), interferon-induced transmembrane protein (IFITM), phenylalanine-4-hydroxylase (PAH), microRNA (MIR), cholesteryl ester transfer protein (CETP), UMP-CMP kinase 2, mitochondrial (CMPK2), radical S-adenosyl methionine domain-containing protein 2 (RSAD2), 3-hydroxyanthranilate 3,4-dioxygenase (HAAO), interferon-induced GTP-binding protein Mx1 (MX1), Ubl carboxyl-terminal hydrolase 18 (USP18), outer dense fiber protein 3B (ODF3B), poly [ADP-ribose] polymerase (PARP), high affinity cAMP-specific 3',5'-cyclic phosphodiesterase 7A (PDE7A), transcription start site (TSS).
