# Supplementary material for: Identification and Characterization of Post-activated B Cells in Systemic Autoimmune Diseases
Source: Front Immunol. 2019 Sep 24;10:2136. doi: 10.3389/fimmu.2019.02136 (PMC6768969; doi:10.3389/fimmu.2019.02136)
Supplement: Supplementary file 11 [file Table_5.DOCX]

# Abbreviations

| Y^352/223/759^ | tyrosine 352, 223 or 759 |
| --- | --- |
| S^473^ | serine 473 |
| AID | autoimmune diseases |
| TLR9 | toll like receptor 9 |
| SLE | Systemic Lupus Erythematosus |
| RA | Rheumatoid Arthritis |
| pSS | primary Sjögren’s Syndrome |
| BCR | B cell receptor |
| ANA | anti-nuclear antibodies |
| dsDNA | double stranded DNA |
| PTK | protein tyrosine kinases |
| PLCγ2 | 1-Phosphatidylinositol-4,5-bisphosphate phosphodiesterase gamma-2 |
| Btk | Bruton's tyrosine kinase |
| Akt1 | protein kinase B |
| ASC | Antibody secreting cells |
| PBMC | Peripheral blood mononuclear cell |
| GWAS | Genome wide association studies |
| EWAS | Epigenome wide association studies |
| DMCT | Dunnett’s test for multiple comparisons |
| BMCT | Bonferroni’s test for multiple comparisons |
| PTP | Protein tyrosine phosphatase |
| PSP | Protein serine/threonine phosphatase |
| RPTP | Receptor type PTP |
| NRPTP | Non receptor type PTP |
